# Identify Non-mutational p53 Functional Deficiency in Human Cancers

**DOI:** 10.1093/gpbjnl/qzae064

**Published:** 2024-09-26

**Authors:** Qianpeng Li, Yang Zhang, Sicheng Luo, Zhang Zhang, Ann L Oberg, David E Kozono, Hua Lu, Jann N Sarkaria, Lina Ma, Liguo Wang

**Affiliations:** National Genomics Data Center, China National Center for Bioinformation, Beijing 100101, China; Beijing Institute of Genomics, Chinese Academy of Sciences, Beijing 100101, China; University of Chinese Academy of Sciences, Beijing 100049, China; National Genomics Data Center, China National Center for Bioinformation, Beijing 100101, China; Beijing Institute of Genomics, Chinese Academy of Sciences, Beijing 100101, China; University of Chinese Academy of Sciences, Beijing 100049, China; National Genomics Data Center, China National Center for Bioinformation, Beijing 100101, China; Beijing Institute of Genomics, Chinese Academy of Sciences, Beijing 100101, China; University of Chinese Academy of Sciences, Beijing 100049, China; National Genomics Data Center, China National Center for Bioinformation, Beijing 100101, China; Beijing Institute of Genomics, Chinese Academy of Sciences, Beijing 100101, China; University of Chinese Academy of Sciences, Beijing 100049, China; Division of Computational Biology, Mayo Clinic College of Medicine and Science, Rochester, MN 55905, USA; Department of Radiation Oncology, Dana-Farber Cancer Institute and Brigham and Women’s Hospital, Boston, MA 02215, USA; Department of Biochemistry & Molecular Biology and Tulane Cancer Center, Tulane University School of Medicine, New Orleans, LA 70112, USA; Department of Radiation Oncology, Mayo Clinic College of Medicine and Science, Rochester, MN 55905, USA; National Genomics Data Center, China National Center for Bioinformation, Beijing 100101, China; Beijing Institute of Genomics, Chinese Academy of Sciences, Beijing 100101, China; University of Chinese Academy of Sciences, Beijing 100049, China; Division of Computational Biology, Mayo Clinic College of Medicine and Science, Rochester, MN 55905, USA; Department of Biochemistry and Molecular Biology, Mayo Clinic College of Medicine and Science, Rochester, MN 55905, USA; Bioinformatics and Computational Biology Graduate Program, University of Minnesota Rochester, Rochester, MN 55904, USA

**Keywords:** p53 deficiency, Cancer, DNA mutation, Composite expression, Machine learning

## Abstract

An accurate assessment of p53’s functional statuses is critical for cancer genomic medicine. However, there is a significant challenge in identifying tumors with non-mutational p53 inactivation which is not detectable through DNA sequencing. These undetected cases are often misclassified as p53-normal, leading to inaccurate prognosis and downstream association analyses. To address this issue, we built the support vector machine (SVM) models to systematically reassess p53’s functional statuses in *TP53* wild-type (*TP53*^WT^) tumors from multiple The Cancer Genome Atlas (TCGA) cohorts. Cross-validation demonstrated the good performance of the SVM models with a mean area under the receiver operating characteristic curve (AUROC) of 0.9822, precision of 0.9747, and recall of 0.9784. Our study revealed that a significant proportion (87%–99%) of *TP53*^WT^ tumors actually had compromised p53 function. Additional analyses uncovered that these genetically intact but functionally impaired (termed as predictively reduced function of p53 or *TP53*^WT^-pRF) tumors exhibited genomic and pathophysiologic features akin to *TP53*-mutant tumors: heightened genomic instability and elevated levels of hypoxia. Clinically, patients with *TP53*^WT^-pRF tumors experienced significantly shortened overall survival or progression-free survival compared to those with predictively normal function of p53 (*TP53*^WT^-pN) tumors, and these patients also displayed increased sensitivity to platinum-based chemotherapy and radiation therapy.

## Introduction

Genetic alterations resulting in the gain or loss of gene function are major drivers of cancer development, and the identification of genomic variants through tumor DNA sequencing has significantly advanced our understanding of cancer genetics [1–3]. The most representative example is the tumor suppressor p53 (encoded by the *TP53* gene), a transcription factor that plays a critical role in preventing tumorigenesis and tumor progression [4–6]. *TP53* is the most frequently mutated gene that undergoes genetic inactivation in at least 20 different types of cancer [7]. Somatic mutation frequencies of *TP53* exceed 50% in ovarian, esophageal, pancreatic, lung, colorectal, uterine, head and neck, oral (gingivobuccal), soft tissue (leiomyosarcoma), gastric, and biliary tract cancers [International Cancer Genome Consortium (ICGC): https://dcc.icgc.org/genes/ENSG00000141510/mutations]. As a result, *TP53* is one of the most extensively studied genes, and the transcriptional targets of p53 are well characterized [8]. The functional activities of p53 have been frequently associated with the response to chemotherapy and radiation therapy (RT) [9–14]. Moreover, p53 dysfunction has been linked to immunosuppression and immune evasion [15–17]. Therefore, accurately assessing the functional statuses of p53 is critical for prognosis and personalized medicine.

However, genetic alteration is not the sole mechanism responsible for disrupting or activating protein function; there is a growing recognition that p53 function can be inactivated even in tumors with genetically wild-type *TP53*. While sporadic studies have provided some support for this notion [18–24], a systematic evaluation using large clinical samples is lacking. On the other hand, the underlying mechanisms may involve various non-mutational factors such as post-translational modifications [25–29], DNA methylation [30], chromatin states [31], and interplaying with microRNAs [32,33]. These molecular mechanisms are widespread, but their heterogeneous nature presents a formidable challenge in characterizing them. Hence, a reevaluation of p53 inactivation that goes beyond *TP53* mutations not only provides a more holistic comprehension of p53’s involvement in cancer biology but also paves the way for the development of p53-targeted therapies.

In this study, our objective is to reassess the functional statuses of p53 in tumors with wild-type *TP53.* We hypothesized that both mutational and non-mutational inactivation of p53 could be reflected by the altered expression of genes regulated by p53. Toward this end, we first defined cancer type-specific gene sets (encompassing both direct and indirect targets of p53), which can serve as indicators of p53’s functional statuses. This was accomplished through a systematic literature review and differential expression analysis (DEA) of RNA sequencing (RNA-seq) data. We then calculated the composite expression score (CES) derived from these genes using various algorithms, including gene set variation analysis (GSVA) [34], single sample gene set enrichment analysis (ssGSEA) [35], combined Z-score [36,37], and the first principal component (PC1) of principal component analysis (PCA). Next, we trained and validated support vector machine (SVM) models using CESs from non-cancerous adjacent normal tissues (referred to as the “NT” group, assumed to have normal p53 function) and tumor samples harboring *TP53* truncating mutations (referred to as the “*TP53*^TM^” group, assumed to have lost or reduced p53’s tumor suppressor function). These SVM models were applied to tumors with wild-type *TP53* (referred to as the “*TP53*^WT^” group) and tumors with *TP53* missense/in-frame mutations (referred to as the “*TP53*^MM^” group). Finally, we systematically evaluated our predictions using multi-omics and clinical data.

## Results

### Identification of cancer-specific gene sets reflecting p53’s functional statuses in seven cancer types

**Figure 1:**
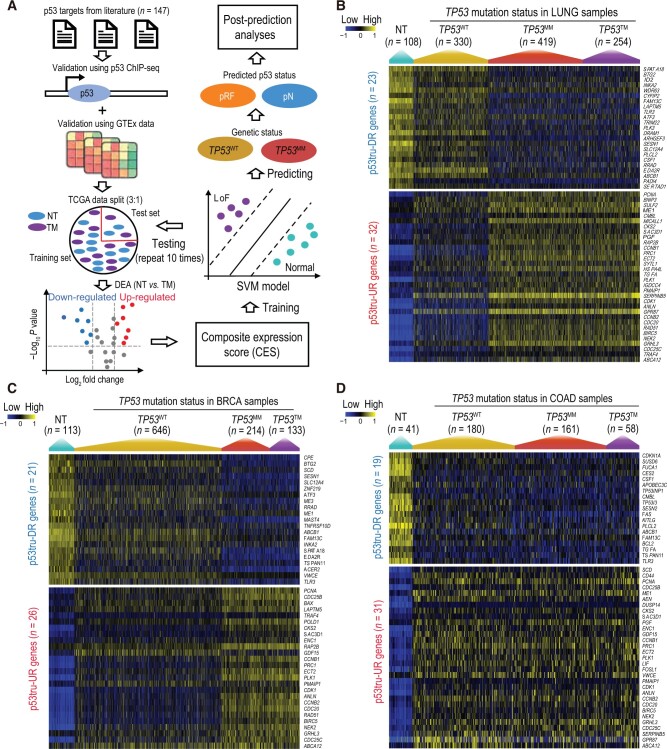
Identification of p53-regulated genes and their expression profiles in TCGA lung, breast, and colon cancers **A**. Analytic workflow depicting the process to identify p53tru-DR and p53tru-UR genes as well as train and validate the SVM models. **B**.–**D**. Heatmaps illustrating the expression profiles of p53tru-DR and p53tru-UR genes in TCGA LUNG (B), BRCA (C), and COAD (D) cohorts. The samples within each cohort were categorized into four groups based on TCGA designation: NT, *TP53*^WT^, *TP53*^MM^, and *TP53*^TM^. CES, composite expression score; ChIP-seq, chromatin immunoprecipitation followed by sequencing; DEA, differential expression analysis; GTEx, Genotype-Tissue Expression; pRF, predicted reduced function; pN, predicted normal; SVM, support vector machine; TCGA, The Cancer Genome Atlas; NT, adjacent normal tissue; *TP53*^WT^, tumor tissue with wild-type *TP53*; *TP53*^MM^, tumor tissue with *TP53* missense or in-frame mutations; *TP53*^TM^, tumor tissue with *TP53* truncating mutations; p53tru-DR gene, gene with down-regulated expression upon p53 truncation; p53tru-UR gene, gene with up-regulated expression upon p53 truncation; LoF, p53 loss of function; LUNG, lung cancer; BRCA, breast cancer; COAD, colon cancer.

We identified a set of p53-regulated genes whose expression levels are statistically associated with p53 truncation for each cancer type and used them as features to predict the functional statuses of p53 in these cancers. First, we compiled a list of 147 genes, comprising both direct and indirect targets, from a comprehensive study conducted by Fischer [8]. We examined the expression profiles of these 147 genes using RNA-seq data from the Genotype-Tissue Expression (GTEx) datasets and assessed the p53 bindings using the chromatin immunoprecipitation followed by sequencing (ChIP-seq) data from the ReMap2020 database [38] (**Figure 1**A). These genes were expressed across various normal tissues, and their promoters displayed evidence of p53 binding (Table S1). To further validate these p53-regulated genes, we reanalyzed RNA-seq and ChIP-seq data obtained from MCF-7 cells that possess wild-type *TP53*, both before and after p53 activation by gamma irradiation. We observed minimal or weak p53 bindings at the promoters of p53-regulated genes (such as *ATF3* and *BTG2*) prior to p53 activation. However, upon p53 activation, significant p53 bindings were observed, accompanied by consistent up-regulation in the expression levels of these genes [39] (Figure S1).

The transcriptional programs regulated by p53 vary by tissue [40–42]. To define a gene set that can serve as features for predicting p53’s functional statuses in each selected cancer type, we conducted gene expression analyses between NT and *TP53*^TM^ groups. This analysis was performed using The Cancer Genome Atlas (TCGA) RNA-seq data for the previously selected 147 genes in each cancer type (Figure 1A). As a result, we identified a set of genes that were down-regulated (referred to as p53tru-DR genes) and a set of genes that were up-regulated (referred to as p53tru-UR genes) in the *TP53*^TM^ group for each specific cancer type. It should be noted that p53tru-UR genes primarily represent genes whose expression is negatively regulated by normal p53, and the underlying mechanisms are likely indirect and not yet fully understood [8]. Finally, we identified 55, 47, 50, 39, 48, 58, and 50 genes regulated by p53 for TCGA lung cancer (LUNG), breast cancer (BRCA), colon cancer (COAD), head and neck cancer (HNSC), stomach cancer (STAD), endometrioid cancer (UCEC), and liver cancer (LIHC) cohorts, respectively. Among them, 12 genes were shared across all seven cancer types (Table S1). It is worth noting that we focused on identifying p53-regulated genes and building SVM models for these seven TCGA cancer types because each of them has sufficient non-cancerous NT samples (*n* > 30) and *TP53*^TM^ samples (*n* > 30) for training and testing. Additionally, we reanalyzed an independent lung adenocarcinoma (LUAD) dataset of East Asians to verify the selected gene set (see Materials and methods). We identified 40 (out of 147 candidates) p53-regulated genes in this dataset using the same method. First, a high degree (37 or 92.5%) of the genes overlapped with p53-regulated genes identified in the TCGA LUNG cohort and showed consistent up-regulated and down-regulated direction. Second, using the p53-regulated genes identified in the TCGA LUNG cohort, we performed unsupervised clustering to assess their potential representation of the p53’s functional statuses (NT and TM) in this East Asian dataset. We found that all of the *TP53*^TM^ samples were predicted to be predictively reduced function of p53 (pRF), and 98.8% of the NT samples were predicted to be predictively normal function of p53 (pN). These results demonstrate the robustness of our gene selection strategy.

### Characterizing composite expression of p53tru-DR and p53tru-UR genes

We analyzed the expression profiles of p53tru-DR and p53tru-UR genes across four distinct groups: NT, *TP53*^WT^, *TP53*^MM^, and *TP53*^TM^ in TCGA LUNG, BRCA, and COAD cohorts. As anticipated, p53tru-DR and p53tru-UR genes exhibited the opposite trends; the expression of p53tru-DR genes was significantly decreased in the *TP53*^TM^ group, while the expression of p53tru-UR genes was significantly increased, which is consistent with the compromised p53 status in this group (Figure 1B–D). Interestingly, similar expression patterns of these p53-regulated genes were observed in the *TP53*^MM^ group (Figure 1B–D). This suggests impaired p53 function in the *TP53*^MM^ samples and indicates that missense/in-frame mutations and truncating mutations have a comparable impact on p53’s cellular activity. This observation aligns with the fact that most missense mutations occur within the DNA-binding domain, leading to the disruption of p53’s ability to bind to DNA and transactivate its downstream targets [43]. These findings collectively indicate a reduced tumor suppressor function of p53 in *TP53*^WT^ samples.

Our hypothesis is that the impaired p53 function could be predicted by the altered expression of its regulated genes, regardless of its mutational status. To characterize the overall impact of a set of genes, we calculated the CESs of p53tru-DR and p53tru-UR genes using four different algorithms, including GSVA [34], ssGSEA [35], combined Z-score [36,37], and PCA (Table S2). Compared to the expression level of individual genes, the CES not only provides a combined and stable measure of p53 activity but also reduces the dimensionality of the expression data and helps mitigate potential overfitting of the SVM model.

**Figure 2:**
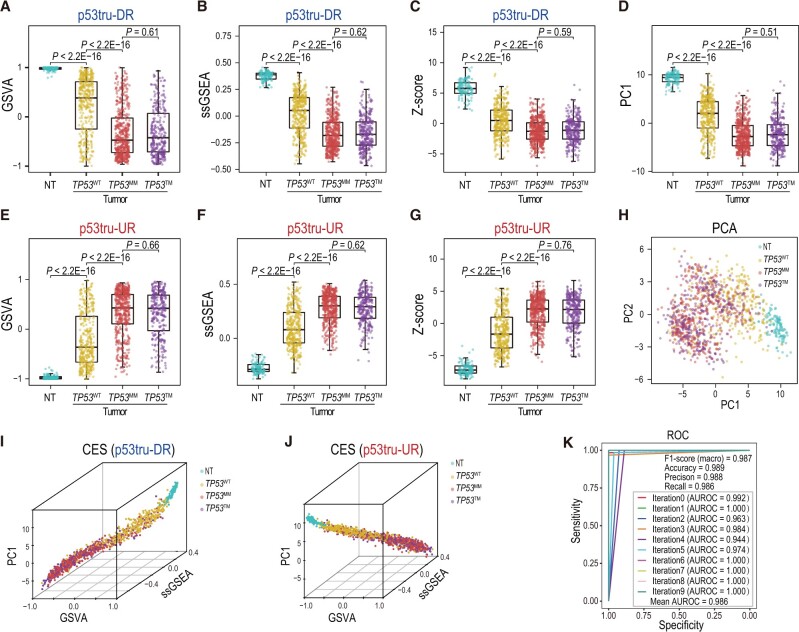
CESs and ROC curve of the SVM model built from the TCGA LUNG cohort **A**.–**C**. CESs of p53tru-DR genes calculated by GSVA (A), ssGSEA (B), and combined Z-score (C) methods. In each panel, the blue, yellow, red, and purple box-and-whisker plots indicate the distribution of CESs in NT, *TP53*^WT^, *TP53*^MM^, and *TP53*^TM^ groups, respectively. **D**. CESs calculated by the PC1 scores combining p53tru-DR and p53tru-UR genes. **E**.–**G**. CESs of p53tru-UR genes calculated by GSVA (E), ssGSEA (F), and combined Z-score (G) methods. **H**. Two-dimensional PCA plot demonstrating the clusters of NT, *TP53*^WT^, *TP53*^MM^, and *TP53*^TM^ samples. **I**. and** J**. Three-dimensional scatter plots illustrating the combinatorial effects of CESs calculated from p53tru-DR genes (I) and p53tru-UR genes (J), respectively. **K**. Performance evaluation of the SVM model using ten-time hold-out validation. GSVA, gene set variation analysis; ssGSEA, single-sample gene set enrichment analysis; PCA, principal component analysis; PC1, first principal component; PC2, second principal component; ROC, receiver operating characteristic; AUROC, area under the receiver operating characteristic curve.

Using the LUNG dataset as an example, we observed significant inverse correlations between CESs calculated from p53tru-DR and p53tru-UR genes. The Pearson’s correlation coefficients were −0.996, −0.998, and −0.749 for GSVA, ssGSEA, and combined Z-score, respectively (Figure S2A–C), indicating that both p53tru-DR and p53tru-UR genes could equally reflect p53’s functional statuses. In addition, we observed significant positive correlations (Pearson’s correlation coefficients ranged from 0.874 to 0.996) among CESs calculated from p53tru-DR or p53tru-UR genes by different algorithms, indicating excellent concordances among these algorithms (Figure S2D–I). When comparing the CESs across the NT, *TP53*^WT^, *TP53*^MM^, and *TP53*^TM^ groups, we found that the CESs of the NT samples tightly congregated within a narrow range. In contrast, the CESs of the tumor samples (including *TP53*^WT^, *TP53*^MM^, and *TP53*^TM^ groups) showed a higher degree of dispersion, suggesting the increased heterogeneity in p53 activity among tumor samples (Figure S3). Consistent with the gene-level expression data (Figure 1B), the CESs of *TP53*^WT^ samples were intermediate between those of NT and *TP53*^TM^ groups, while the CESs of the *TP53*^MM^ samples resembled those of the *TP53*^TM^ samples (**Figure 2**A–J).

### Building SVM models to predict p53 functional statuses in different cancer types

We proceeded to train SVM models using seven CESs calculated using four different algorithms, with the purpose of predicting p53’s functional statuses in *TP53*^WT^ and *TP53*^MM^ tumors. To achieve the best performance, these models were independently trained and validated for each cancer type (see Materials and methods). Our cross-validation results exhibited outstanding performance by these SVM models. As exemplified by the LUNG cohort, the mean precision, recall, F1-score, and area under the receiver operating characteristic curve (AUROC) were 0.988, 0.986, 0.987, and 0.986, respectively (Figure 2K). Similar performance was achieved in the remaining six cancer types (Table S3).

The limited number of NT or *TP53*^TM^ samples in other TCGA cancer types prevented us from training separate SVM models for these cancer types. Instead, we created a pan-cancer cohort and trained an SVM model by combining nine cancer types [LUNG, BRCA, COAD, HNSC, STAD, UCEC, LIHC, bladder cancer (BLCA), esophageal cancer (ESCA); *n* = 5160] with a relatively higher number of NT samples (*n* > 10) and *TP53*^TM^ samples (*n* > 30) (Figure S4). Despite the intrinsic heterogeneity of different cancer types, the pan-cancer SVM model demonstrated outstanding performance (Figure S4B; Table S3). However, it should be noted that building a pan-caner SMV model is a compromise approach when individual cancer types lack sufficient samples to train their own SVM models, as it might be dominated by a few cancer types with exceptionally larger sample sizes, potentially introducing bias.

### Prevalent non-mutational inactivation of p53 in human cancers predicted using SVM models

**Figure 3:**
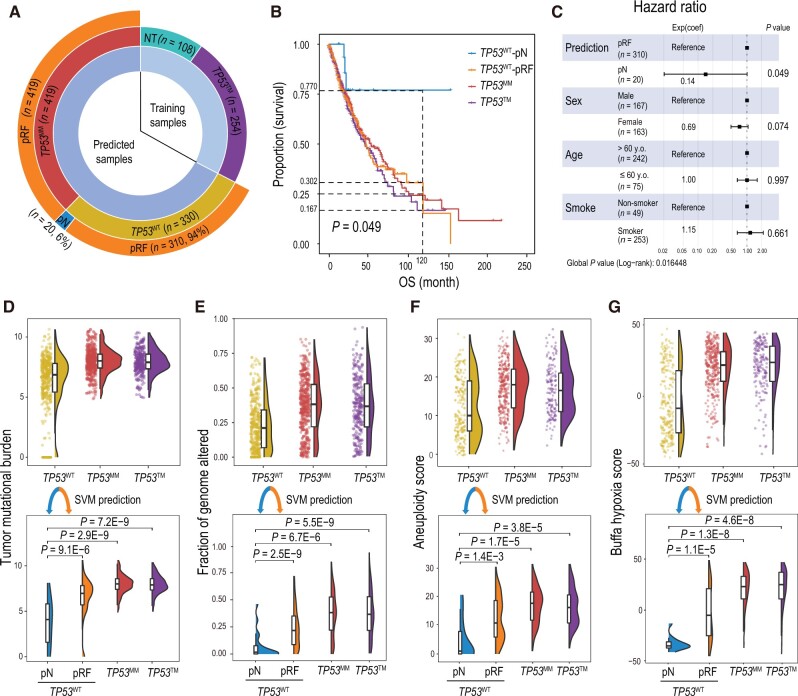
Evaluation of the SVM predictions in the TCGA LUNG cohort **A**. A sunburst chart showing the breakdown of the LUNG samples before (inner and middle layer circles) and after (outer layer circle) SVM prediction. Training samples consist of NT and *TP53*^TM^ groups, representing p53-normal and p53-LoF statuses, respectively. Samples that were subjected to SVM prediction include *TP53*^WT^ and *TP53*^MM^ groups. Samples in *TP53*^WT^ and *TP53*^MM^ groups were predicted as normal (pN) or reduced function (pRF) by the SVM model. **B**. OS of patients with *TP53*^MM^, *TP53*^TM^, *TP53*^WT^-pN, and *TP53*^WT^-pRF tumors. **C**. Forest plot of a Cox proportional hazards model showing *P* values, hazard ratios, and the 95% confidence intervals for covariates. **D**. Upper panel: comparison of tumor mutational burden among *TP53*^WT^, *TP53*^MM^, and *TP53*^TM^ groups. Lower panel: the *TP53*^WT^ tumors were further divided into two subgroups (*TP53*^WT^-pN and *TP53*^WT^-pRF) based on SVM prediction. **E**. Upper panel: comparison of the copy number variation burden measured by “Fraction of genome altered” among *TP53*^WT^, *TP53*^MM^, and *TP53*^TM^ groups. Lower panel: the *TP53*^WT^ tumors were further divided into two subgroups (*TP53*^WT^-pN and *TP53*^WT^-pRF) based on SVM prediction. **F**. Upper panel: comparison of the aneuploidy scores among *TP53*^WT^, *TP53*^MM^, and *TP53*^TM^ groups. Lower panel: the *TP53*^WT^ tumors were further divided into two subgroups (*TP53*^WT^-pN and *TP53*^WT^-pRF) based on SVM prediction. **G**. Upper panel: comparison of Buffa hypoxia scores among *TP53*^WT^, *TP53*^MM^, and *TP53*^TM^ groups. Lower panel: the *TP53*^WT^ tumors were further divided into two subgroups (*TP53*^WT^-pN and *TP53*^WT^-pRF) based on SVM prediction. *TP53*^WT^-pN, tumor tissue with wild-type *TP53* predicted as normal; *TP53*^WT^-pRF, tumor tissue with wild-type *TP53* predicted as reduced function; OS, overall survival.

We examined the predictive power of our SVM models by applying them to *TP53*^WT^ and *TP53*^MM^ tumors, which were excluded from the training and testing phases. To avoid potential confusion caused by the term “p53 loss of function (LoF)”, which typically refers to p53 dysfunction due to mutations, we designated samples predicted to be *TP53*^TM^-like samples as “reduced function (RF)” instead. The results revealed that the majority of *TP53*^WT^ samples (87%–99%) and almost all *TP53*^MM^ samples (98%–100%) were predicted as RF in all seven cohorts: LUNG (94%, 100%), BRCA (87%, 98%), COAD (99%, 100%), HNSC (96%, 100%), STAD (96%, 98.17%), UCEC (97%, 99%), and LIHC (90%, 100%) (Table S4). Using the LUNG cohort as an example, 94% (310 out of 330) of the *TP53*^WT^ samples and 100% (*n* = 419) of *TP53*^MM^ samples were predicted to be RF (**Figure 3**A**)**. These results indicate the prevalence of non-mutational inactivation of p53 in human cancers.

To assess the prognostic value of the SVM prediction, we analyzed the LUNG cohort by dividing *TP53*^WT^ tumors into two subgroups: *TP53*^WT^-pRF, representing *TP53*^WT^ samples predicted as “reduced p53 function”, and *TP53*^WT^-pN, representing *TP53*^WT^ samples predicted as “normal p53 function”. Our analysis revealed that the 10-year overall survival (OS) rates for *TP53*^WT^-pN (*n* = 20) and *TP53*^WT^-pRF (*n* = 310) were 77.0% and 30.2%, respectively. As reference points, the 10-year OS rates for *TP53*^MM^ (*n* = 419) and *TP53*^TM^ (*n* = 254) were 24.3% and 16.7%, respectively (Figure 3B). It is noted that the difference in OS rates between LUNG patients with *TP53*^WT^-pN tumors and those with *TP53*^WT^-pRF tumors became more significant after adjusting for demographic variables such as sex, age, and smoking status using Cox regression (*P* = 0.016) (Figure 3C). In contrast, the OS rate of patients with *TP53*^WT^-pRF tumors did not differ significantly from that of patients with *TP53*^MM^ or *TP53*^TM^ tumors. The TCGA BRCA was divided into five subtypes, including “Basal”, “HER2+”, “Luminal-A”, “Luminal-B”, and “Normal-like” (Figure S5A–F). Most (71 out of 85 or 83.5%) of the *TP53*^WT^-pN tumors were classified as Luminal-A subtype, but we did not observe a significant difference in progression-free survival (PFS) of patients within this subtype (Figure S5D). The remaining 15.3% (13 out of 85) of *TP53*^WT^-pN cases are normal-like BRCA tumors. Within this subgroup, the *TP53*^WT^-pRF patients exhibited a significantly reduced PFS compared to the *TP53*^WT^-pN group (*P* = 0.017, log-rank test) (Figure S5F). We were unable to perform comparative analyses for “Basal”, “HER2+”, and “Luminal-B” subtypes as there were almost no *TP53*^WT^-pN cases. Normal-like tumors were generally considered “artifacts” resulting from a high percentage of normal specimens or slow-growing basal-like tumors [44]. However, our data suggest that the PFS is significantly reduced when p53 function is compromised, supporting the classification of normal-like tumors as a distinct subtype rather than mere normal tissue contamination. In summary, in both lung and breast (normal-like) cancers, *TP53*^WT^-pRF tumors exhibit a significantly worse prognosis compared to the *TP53*^WT^-pN tumors.

Considering the crucial role of p53 in DNA damage repair [45,46], we hypothesized that p53-defective tumors would accumulate more DNA damage compared to p53-normal tumors. To investigate this hypothesis, we compared measurements of genome and chromosome instability, including tumor mutational burden, copy number variation burden, and aneuploidy score, between the *TP53*^WT^-pRF and *TP53*^WT^-pN groups. The analysis revealed significantly higher levels of genome instability in the *TP53*^WT^-pRF group compared to the *TP53*^WT^-pN group, indicating deficient DNA damage repair in *TP53*^WT^-pRF tumors (Figure 3D–F). Similar trends were observed in the other TCGA cohorts (Table S5). It is known that p53 can decrease cell hypoxia (*i.e.*, insufficient oxygen in the tumor microenvironment) by inhibiting HIF1A activity [47–49]. Consistent with these findings, the Buffa hypoxia score [50] was significantly lower in the *TP53*^WT^-pN tumors compared to that in the *TP53*^WT^-pRF, *TP53*^MM^, and *TP53*^TM^ tumors (Figure 3G). Overall, *TP53*^WT^-pRF tumors exhibited increased genomic instability, worse prognosis, and higher hypoxia levels, resembling the *TP53*-mutant tumors. These data not only functionally reaffirm our SVM predictions but also reveal the potential limitation of determining *TP53* status based solely on DNA sequencing.

### Increased sensitivity of *TP53*^WT^-pRF tumors to chemotherapy and RT

The impact of mutant *TP53* on the response to chemotherapy and RT remains controversial. Several studies have associated *TP53* mutations with reduced sensitivity to these therapies [10,11,51–53], while other studies have suggested that p53 inactivation actually increases the tumor’s sensitivity [12–14]. Unfortunately, direct comparisons of therapy efficacy using TCGA samples are not feasible due to the lack of treatment response data. To overcome this limitation, we assessed the chemosensitivity and radiation sensitivity using previously published gene signatures (Table S6) and conducted investigations with preclinical animal models to examine the therapeutic effects of radiation. Specifically, we evaluated chemosensitivity in LUNG and BRCA cohorts using recombination proficiency score (RPS) which has been clinically validated in BRCA and non-small cell lung cancer (NSCLC) patients [54]. For the assessment of tumor radiation sensitivity, we utilized the radiation sensitivity signature (RSS) score and tested it in BRCA [55] (see Materials and methods). To explore the relationship between the predicted p53 status and the response to RT *in vivo*, we analyzed an independent dataset consisting of 35 patient-derived xenograft (PDX) models that closely mimic the genetic and phenotypic characteristics of glioblastoma (GBM) patients [56].

**Figure 4:**
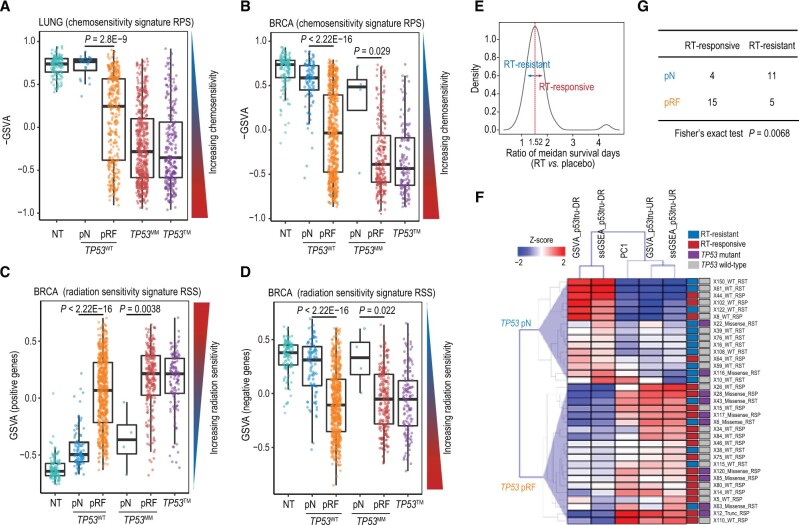
Relationships between p53 statuses and chemotherapy and RT sensitivities **A**. Comparison of the RPSs amongst the NT, *TP53*^WT^-pN, *TP53*^WT^-pRF, *TP53*^MM^, and *TP53*^TM^ groups in the TCGA LUNG cohort. **B**. Comparison of the RPSs amongst the NT, *TP53*^WT^-pN, *TP53*^WT^-pRF, *TP53*^MM^-pN, *TP53*^MM^-pRF, and *TP53*^TM^ groups in the TCGA BRCA cohort. The chemosensitivity signature RPS is represented by negative GSVA score (see Materials and methods). **C**. and** D**. Comparison of the RSS scores of positive (C) and negative (D) genes amongst NT, *TP53*^WT^-pN, *TP53*^WT^-pRF, *TP53*^MM^-pN, *TP53*^MM^-pRF, and *TP53*^TM^ groups in the BRCA cohort, respectively. The radiation sensitivity signature RSS is represented by GSVA score (see Materials and methods). **E**. Distribution of the ratio of median survival days between the RT group and the placebo group. PDX models with ratios ≥ 1.52 were considered RT-responsive. **F**. A heatmap showing the p53 functional statuses predicted by unsupervised clustering using composite expression (GSVA, ssGSEA, and PC1) of GBM-specific p53tru-DR and p53tru-UR genes in PDX data. RT response and *TP53* mutation status of each sample are color-coded on the right side of the heatmap. **G**. A contingency table shows that pRF samples are significantly associated with RT responsiveness. RPS, recombination proficiency score; RSS, radiation sensitivity signature; RT, radiation therapy; GBM, glioblastoma; PDX, patient-derived xenograft.

When comparing chemosensitivity across groups with different p53 statuses in the LUNG cohort, we found that samples in the NT group exhibited the highest RPSs, while tumors in the *TP53*^MM^ and *TP53*^TM^ groups displayed the lowest RPSs. Expectedly, the RPSs of *TP53*^WT^-pN tumors were close to those of the NT group and were significantly higher than those of the *TP53*^WT^-pRF tumors (*P* = 2.8E−9, two-sided Wilcoxon test) (**Figure 4**A). Similar results were observed in the BRCA cohort. Notably, four BRCA *TP53*^MM^ tumors that were predicted to be p53 normal also had significantly higher RPSs than the remaining *TP53*^MM^ samples (*P* = 0.029, two-sided Wilcoxon test) (Figure 4B). The lower RPS was linked to increased mutagenesis, adverse clinical features, and inferior patient survival rates, but such adverse prognosis could be counteracted by adjuvant platinum-based chemotherapy [54]. While mutations in *BRCA1*/*BRCA2* are commonly known as the primary drivers of homologous recombination deficiency (HRD), strong positive associations between the *TP53* mutation ratio and HRD scores have been observed in TCGA pan-cancer analysis [57]. Moreover, studies have indicated the involvement of p53 in regulating homologous recombination [58,59]. These data suggest that platinum-based chemotherapy may provide greater benefits to *TP53*^WT^-pRF tumors compared to *TP53*^WT^-pN tumors.

We assessed radiation sensitivity by dividing the RSS gene signature into positive (*i.e.*, genes positively correlated with radiation sensitivity) and negative (*i.e.*, genes negatively correlated with radiation sensitivity) subsets. Our analysis focused on the TCGA BRCA cohort since the RSS signature was derived from BRCA. When comparing *TP53*^WT^-pN tumors with *TP53*^WT^-pRF tumors using positive genes, we found a significant increase in RSS scores for *TP53*^WT^-pRF tumors (Figure 4C), indicating heightened radiation sensitivity. Meanwhile, when measured using negative genes, *TP53*^WT^-pRF tumors showed significantly decreased RSS scores, also suggesting increased radiation sensitivity (Figure 4D). On the other hand, *TP53*^MM^-pN tumors displayed significantly reduced radiation sensitivity compared to *TP53*^MM^-pRF tumors (Figure 4C and D).

Although there is one gene (*RAD51*) from the RPS signature and four genes from the RSS signature that overlapped with the SVM feature genes for LUNG or BRCA (Figure S6A), we obtained consistent results even after excluding these overlapping genes (Figure S6B–E). These findings collectively indicate that tumors predicted to have reduced p53 function, akin to *TP53*-mutant tumors, show increased sensitivity to platinum-based chemotherapy and RT. This could be attributed to compromised DNA damage repair due to reduced p53 function, making tumor cells more susceptible to the effects of chemotherapy and RT.

We further confirmed this finding *in vivo* using 35 PDX models. We first defined 17 p53tru-DR and 19 p53tru-UR genes from the TCGA GBM cohort (Table S1). Due to limited sample sizes of NT and *TP53*^TM^ samples, training an SVM model to predict p53 statuses was unfeasible. Alternatively, we employed unsupervised clustering to predict the p53 functional statuses using CESs calculated from RNA-seq data of PDX mouse models (Table S7). To determine the RT responsiveness, we calculated the ratio of median survival days between the RT group and the placebo group, and PDX models with ratios ≥ 1.52 were considered as RT-responsive (Figure 4E; Table S7). These results indicate a significant over-representation of RT responders within the pRF group (Fisher’s exact test, *P* = 0.0068) (Figure 4F and G). Although *TP53* mutations were enriched in the pRF group (Figure 4F**)**, the association between *TP53* genetic mutation and RT response did not reach statistical significance (Fisher’s exact test, *P* = 0.13). In summary, these findings suggest that our *in silico* predictions effectively uncover a significant association between p53 status and RT response, which would have been overlooked if solely examining the *TP53* genetic status.

### *TP53* ^WT^-pRF partially attributes to false-negative mutation calling and *MDM2/MDM4* amplification

Next, we sought to explore the potential factors and mechanisms that could account for the majority of *TP53*^WT^ tumors being predicted as RF by the SVM model. We investigated the RNA and protein expression, reevaluated all the *TP53* missense mutations previously reported from whole-exome sequencing (WES) using RNA-seq data, and examined the alteration statuses of the p53 upstream regulators MDM2 and MDM4.

We did not detect significant changes in p53 protein abundance between *TP53*^WT^-pN and *TP53*^WT^-pRF tumors. *TP53* RNA expression levels were even increased in the *TP53*^WT^-pRF tumors (Figure S7), which aligns with previous research indicating that mutant p53 is associated with increased *TP53* messenger RNA (mRNA) expression [60]. These results also suggest that the impaired p53 function observed in the *TP53*^WT^-pRF group is unlikely to be attributed to the reduced *TP53* mRNA and protein levels.

**Figure 5:**
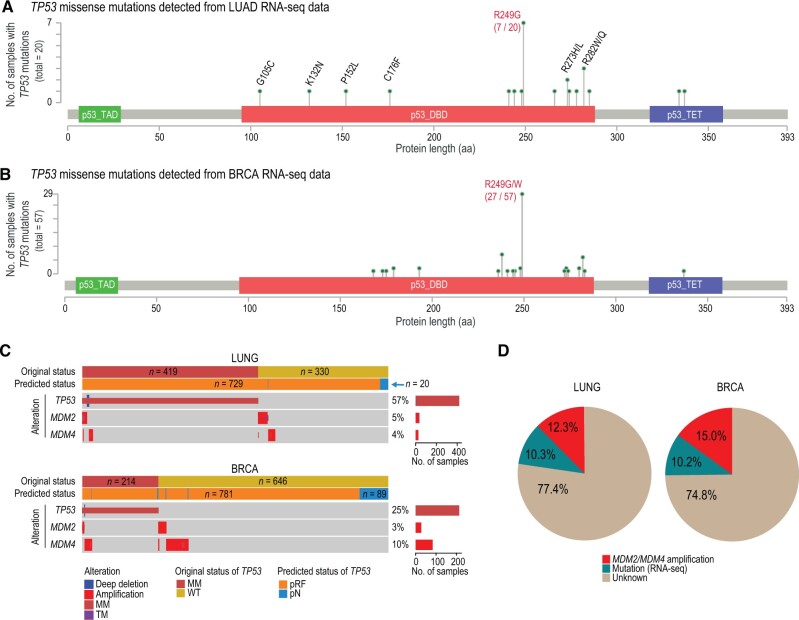
Dissection of *TP53*^WT^-pRF cases **A**. and** B**. Lollipop charts showing the amino acid positions of *TP53* missense mutations identified using RNA-seq data but missed from the WES data in TCGA LUAD (A) and BRCA (B), respectively. The colored boxes indicate the key domains in p53, including TAD (green), DBD (red), and TET (purple). **C**. Oncoprint plot showing the *TP53*’s original and predicted statuses, compared with the amplification statuses of *MDM2* and *MDM4*. Percentages of samples with *TP53*/*MDM2*/*MDM4* alterations among the prediction set (*TP53*^MM^ and *TP53*^WT^ samples) are indicated on the right side, as well as the numbers of these samples (indicated by bar plots). **D**. Pie charts showing the fraction of *TP53*^WT^-pRF cases that can be explained by “mutation detected from RNA-seq” (green) and “*MDM2*/*MDM4* amplification” (red). LUAD, lung adenocarcinoma; aa, amino acid; RNA-seq, RNA sequencing; WES, whole-exome sequencing; TAD, transactivation domain; DBD, DNA-binding domain; TET, tetramerization domain.

We then reassessed all the *TP53* missense mutations, such as R249 and R273, in the *TP53*^WT^-pRF tumors using the RNA-seq data of the LUNG and BRCA cohorts. Surprisingly, 10.3% (32 out of 310) LUNG and 10.2% (57 out of 561) BRCA tumors in the *TP53*^WT^-pRF group were indeed *TP53* mutants, which were supported by the substantial number of RNA-seq reads carrying the mutant alleles (Table S8). For example, two LUAD samples (TCGA-55-6987 and TCGA-55-8621), initially classified as *TP53*^WT^ by TCGA, were reevaluated and found to possess mutant allele fractions (MAFs) of 45% (44 out of 98) and 35% (35 out of 100), respectively. Notably, these two mutations were also detectable in the WES data, albeit with much lower numbers of supporting reads (2 and 5 reads, respectively). This discrepancy explains why they were not identified by the TCGA somatic variant caller (Figure S8). The substantial increase in MAFs observed in the RNA-seq data is probably due to the preferential expression of the mutant alleles. Interestingly, when comparing the amino acid locations of missense mutations reported from WES data, these mutations identified through RNA-seq data were significantly enriched (*P* = 1.05E−6, two-sided Fisher’s exact test) at the p53 R249 position in both LUAD and BRCA (**Figure 5**A and B). A missense mutation at the R249 position is generally recognized as a structural mutation that destabilizes the p53 protein [61]. Furthermore, the mutant tumors rescued from the RNA-seq data exhibited similar genomic and pathophysiology characteristics as the *TP53*^MM^ and *TP53*^TM^ tumors (Figure S9).

MDM2 and its homolog MDM4 are well-documented negative regulators of p53 [62–64]. In our analysis, we found that amplification of *MDM2* and/or *MDM4* was mutually exclusive with *TP53* mutations in LUNG (odds ratio = 4.32, *P* = 2.2E−16, χ^2^ test) and BRCA (odds ratio = 1.46, *P* = 2.0E−16, χ^2^ test) (Figure 5C). Consistent with this observation, all samples displaying amplification of *MDM2* and/or *MDM4* (*n* = 38 in LUNG, *n* = 84 in BRCA) were classified as pRF. These samples accounted for 12.3% and 15.0% of all *TP53*^WT^-pRF samples in the LUNG and BRCA cohorts, respectively (Figure 5D). Collectively, 22%–25% of *TP53*^WT^ tumors predicted as *TP53*^WT^-pRF can be attributed to false-negative results of WES assay or amplification of *MDM2* and *MDM4*.

## Discussion

Tumor suppressor genes such as *RB1*, *PTEN*, and *CDKN2A* are generally inactivated through homozygous deletions, resulting in minimal or complete loss of protein expression (https://www.cbioportal.org/). In contrast, *TP53*, unlike other tumor suppressor genes, predominantly undergoes missense mutations that give rise to altered or dysfunctional protein variants (https://www.cbioportal.org/). In addition, the oncogenic role of mutant p53 has been extensively characterized through multiple lines of evidence [65], including the overexpression of mutant p53, accumulation of mutant p53 in the nucleus and the cytoplasm, and functional studies with *in vivo* and *in vitro* systems [66–69]. The primary objective of this study is to investigate the loss or reduction of p53’s tumor suppressor function in tumors that may appear functionally normal due to the absence of *TP53* genetic alterations. Based on the assumption that the tumor suppressor function of p53 is compromised or significantly reduced in *TP53*^TM^ samples but remains normal in NT samples, we trained SVM models using NT samples and *TP53*^TM^ tumor samples with truncating *TP53* mutations that result in shortened protein. However, it is essential to recognize that our training dataset is not without imperfections, given that the p53 statuses of these samples are inferred rather than definitively determined. To be more precise, the predicted outcomes should be termed NT-like or *TP53*^TM^-like. Looking ahead, datasets that encompass paired RNA and DNA sequencing information, coupled with clearly defined p53 statuses are needed to overcome the existing limitations and gain more conclusive insights. Meanwhile, *TP53* missense mutations that were predicted to be functionally reduced (*TP53*^MM^-pRF) should be interpreted as the “reduced tumor suppressor function of p53”. Nevertheless, we could not rule out the possibility that the mutant p53 protein in the *TP53*^MM^-pRF samples might gain oncogenic functions. Additionally, although p53tru-UR genes are negatively regulated by p53 through indirect and unclear mechanisms, we retained these genes for downstream analyses since they can effectively predict p53’s functional status as p53tru-DR genes do. Our strategy allows us to identify tumors with compromised p53 function resulting from multiple mechanisms, and it is plausible that some cases predicted to have reduced p53 function may genuinely represent a loss of function, while others may indicate dysregulation.

Although the expression of p53-regulated genes could be potentially influenced by other factors, our *TP53*^WT^-pRF predictions, as well as the p53-regulated genes defined in this study, are highly specific to *TP53*. We performed the Fisher’s exact test on the prediction results of the LUNG and BRCA cohorts, to assess whether the mutation status (WT or mutant) of other cancer-related genes correlates with the prediction results (pN and pRF) of *TP53*^WT^ and *TP53*^MM^ samples. Notably, in LUNG, *TP53* emerged as the most significant gene associated with our prediction, with a considerably lower *P* value compared to the other three genes meeting the threshold of *P* < 0.05. Similarly, in BRCA, *TP53* ranked as the 2nd most significant gene after *CDH1* (both *TP53* and *CDH1* showed considerably lower *P* values compared to the other five genes), indicating a potential synergistic effect between *CDH1* and *TP53* and emphasizing the importance of *TP53* in *TP53*^WT^-pRF prediction. Previous studies did report the somatic co-inactivation and collaboration of *TP53* and *CDH1* in breast and other cancers [70,71]. Additionally, p53tru-UR genes and p53tru-DR genes are highly specific to the p53 pathway, as evidenced by their significant enrichment in this pathway compared to others; the “p53 signaling pathway” was ranked as the most significantly enriched pathway (adjusted *P* = 1.26E−31).

According to our analyses, approximately 22%–25% of *TP53*^WT^ tumors predicted as *TP53*^WT^-pRF can be attributed to false-negative results (*i.e.*, *TP53* mutations that failed to be detected from the WES assay) or amplification of *MDM2* and *MDM4*. However, the underlying mechanisms for the remaining 75%–78% of *TP53*^WT^-pRF tumors are unknown and warrant further investigation. In order to gain more insights, we analyzed the DNA methylation data generated from the Infinium HumanMethylation450 BeadChip array. However, we did not observe significant differences in the DNA methylation patterns among all nine CpG sites within the *TP53* gene (cg02087342, cg06365412, cg10792831, cg12041075, cg12041429, cg13468400, cg16397722, cg18198734, and cg22949073) when comparing *TP53*^WT^-pRF and *TP53*^WT^-pN tumors. This suggests that p53 function may be compromised through other non-mutational mechanisms such as post-translational modifications [25–29], chromatin states [31], and interaction with microRNAs [32,33]. Unfortunately, due to the unavailability of matched data, we are unable to perform specific analyses in these areas. We also investigated the potential contributions of “p53-regulated genes” and “p53 functional partners” in the LUNG cohort and found that up to 54.8% of *TP53*^WT^-pRF samples (which cannot be explained by “undetected *TP53* mutation” or “*MDM2/4* amplification”) have mutations in these genes. However, the mutation frequencies of individual genes are low, and we are unable to assess the statistical significance of their associations with *TP53*^WT^-pRF.

It is noted that our data suggest that platinum-based chemotherapy, rather than all kinds of chemotherapies, may provide greater benefits to *TP53*^WT^-pRF tumors compared to *TP53*^WT^-pN tumors. Using drug imputation data of TCGA patients [71,72], we further examined the treatment response of *TP53*^WT^-pRF samples. In the first study [71], we identified 22 drugs exhibiting elevated sensitivity (Wilcoxon rank sum test, adjusted *P* value < 0.05) in *TP53*^WT^-pRF tumors and 5 drugs showing increased sensitivity (adjusted *P* value < 0.05) in *TP53*^WT^-pN tumors. In the second study [72], Cancer Cell Line Encyclopedia (CCLE) S-model (a model trained by non-hematopoietic cell lines in CCLE, “S” denotes solid tumor) prediction identified 13 drugs with increased sensitivity in *TP53*^WT^-pN tumors; Genomics of Drug Sensitivity in Cancer (GDSC) A-model (a model trained by all the cell lines in GDSC, “A” denotes all tumor types) prediction identified 27 drugs showing increased sensitivity in *TP53*^WT^-pN tumors and 20 drugs exhibiting elevated sensitivity in *TP53*^WT^-pRF tumors. However, there are limited overlap and poor consistency between the two studies. Therefore, chemotherapies should be dissected in detail to inform clinical therapy strategies.

In our analysis, we found no significant differences in tumor purity (measured by immunohistochemistry [73]) between *TP53*^WT^-pN and other tumor samples in the LUNG cohort (*P* = 0.460, Wilcoxon rank sum test) and the BRCA cohort (*P* = 0.066, Wilcox rank sum test). This suggests that the *TP53*^WT^-pN tumors are not associated with lower tumor cellularity. Additionally, we identified four *TP53*^MM^ samples from the BRCA cohort (TCGA-E2-A1B1, TCGA-LD-A74U, TCGA-BH-A1FE, and TCGA-EW-A1P1) that were predicted to have normal p53 function (Table S4, sheet C). We confirmed all reported mutations in these four samples using WES and RNA-seq data and ruled out the possibilities of false positives or cross-sample contaminations. The reasons behind these findings remain unclear, and the limited sample size prevents extensive analysis.

While our analytic procedure is robust and can be applied to other datasets, building an SVM model from scratch requires a large number of NT samples and *TP53*^TM^ tumor samples as training data. When obtaining sufficient training data is not practical, alternative unsupervised machine learning approaches such as *K*-means clustering, semi-supervised learning, PCA, and neural networks could be considered.

Our analyses have revealed an intriguing aspect: the majority of *TP53*^WT^ tumors are genetically intact but exhibit functional deficiency in p53. This discovery highlights an inherent limitation in relying solely on DNA markers for patient stratification and segmentation. It emphasizes the need for the development of “ensemble” approaches that incorporate multi-omics data to capture the full complexity of p53 functionality. By considering multiple layers of molecular information, we can gain a more comprehensive understanding of p53 status and its implications in cancer. Such integrated approaches hold great promise for enhancing our ability to characterize tumors and accurately guide personalized treatment strategies.

## Conclusion

In this study, we employed a novel approach to measure p53 activity by defining p53-regulated genes and calculating their CESs as a surrogate. By training and cross-validating SVM models using CES data from the NT (p53-normal) and *TP53*^TM^ groups (p53-RF), we demonstrated the accuracy and effectiveness of our *in silico* approach. Our comprehensive analysis revealed the prevalence of non-mutational p53 inactivation in human malignancies. Moreover, our analyses unveiled that the predicted *TP53*^WT^-pRF tumors exhibited a comparable level of genomic instability to those harboring genetic *TP53* mutation. This included a significant increase in the number of mutations, copy number alterations, and aneuploidy. Importantly, patients with *TP53*^WT^-pRF tumors showed considerably worse OS rates when compared to those with *TP53*^WT^-pN tumors, highlighting the prognostic value of our prediction. Furthermore, when evaluated using clinically validated signatures, *TP53*^WT^-pRF tumors demonstrated significantly heightened sensitivity to platinum-based chemotherapy and RT. This observation was verified in our preclinical animal models of GBM. Additionally, we explored potential factors contributing to the *TP53*^WT^-pRF classification, such as false-negative mutation calling and amplification of *MDM2* and *MDM4*.

## Materials and methods

### Data collection

TCGA somatic mutation data, as well as the pre-calculated fraction of genome altered (FGA) score, mutation count, aneuploidy score, and Buffa hypoxia score, were downloaded from cBioPortal (https://www.cbioportal.org/). TCGA WES and RNA-seq binary alignment map (BAM) files for BRCA and LUNG were downloaded from the Genomic Data Commons (GDC) (https://portal.gdc.cancer.gov/). TCGA level-3 RNA-seq expression data, demographic data, and survival data were downloaded from the Xena web server (https://xena.ucsc.edu/). Pre-calculated raw RNA-seq read counts were used to identify differentially expressed genes between the NT and *TP53*^TM^ groups. The log_2_-transformed fragments per kilobase of exon per million mapped fragments (FPKM) was used to calculate CESs. Transcript per million (TPM) was used to calculate the GSVA score to evaluate chemosensitivity and radiation sensitivity. ChIP-seq data (p53-binding peaks) were downloaded from the ReMap2020 database (https://remap.univ-amu.fr/). Gene expression data (TPM) of NT samples were downloaded from the GTEx (release V8) data portal (https://gtexportal.org/home/).

### Identification of p53-regulated genes across different cancer types

We first compiled a set of 147 genes that have been experimentally validated as being regulated by p53. This set includes 116 genes identified as directly activated targets of p53 (refer to Table 1 in [8]) and 31 genes that are repressed by p53 by indirect regulation (refer to Table 2 in [8]). We then analyzed p53 ChIP-seq data from ReMap2020 [38] to verify p53 binding within the gene body or the promotor region. The basal level expression of p53-regulated genes in NT samples was evaluated using the RNA-seq data of GTEx [74]. Only genes with median TPM > 1 were included in our downstream analyses. To identify feature genes whose expression could reflect p53’s functional status in each cancer type, we performed DEA for the 147 genes in each cancer by comparing the NT group (adjacent normal tissue samples) to the *TP53*^TM^ group (tumor samples harboring *TP53* truncating mutations) using DESeq2 [75]. The significance threshold was set at an adjusted *P* value ≤ 0.05 and |fold change| ≥ 2. We defined “p53tru-DR” genes as those showing down-regulation in *TP53*^TM^ samples compared to NT samples. Conversely, “p53tru-UR” genes referred to genes showing up-regulation in *TP53*^TM^ samples. To verify our gene set, we included an independent LUAD dataset of East Asians (https://src.gisapps.org/OncoSG_public/study/summary?id=GIS031) into our analysis [76]. This dataset includes 21 *TP53*^TM^ samples and 88 NT samples. Employing the same procedure, we identified differentially expressed genes out of 147 p53 target candidates from this dataset and compared the overlap of p53-regulated genes between this dataset and the TCGA LUNG cohort. Additionally, using the CESs calculated from p53-regulated genes identified in the TCGA LUNG cohort, we predicted the p53’s functional statuses in this independent dataset by unsupervised clustering.

### Computation of CESs

We employed four algorithms to calculate the CES, namely: (1) GSVA [34]; (2) ssGSEA [35]; (3) the PC1 score of the PCA; and (4) combined Z-score [36,37]. To calculate the combined Z-score, gene expression values (log_2_ FPKM) of each sample were converted into Z-scores by Z = (*x* − μ)/σ, where μ and σ is the average and standard deviation of log_2_ FPKM across all samples of a gene. Given a gene set *γ* = {1,⋯,*k*} with standardized values *z*_1_,⋯,*z_k_*, for each gene in a specific sample, the combined Z-score *Z_γ_* for the gene set *γ* is defined as:

(1) $$\begin{array}{c} Z_{\gamma }=\frac{\sum_{i=1}^{k}Z_{i}}{\sqrt{k}} \end{array}$$

GSVA, ssGSEA, and combined Z-score were calculated separately for p53tru-DR and p53tru-UR genes, while PC1 was computed using all p53-regulated genes, which resulted in a total of seven CESs for each sample. The GSVA package (https://www.bioconductor.org/packages/release/bioc/html/GSVA.html) was used to calculate GSVA, ssGSEA, and Z-score. The Python package scikit-learn (https://scikit-learn.org/stable/) was used to perform PCA analyses.

### Training and evaluating the performance of SVM models

SVM models with linear kernel were trained using the CESs of NT (coded as “0”) and *TP53*^TM^ (coded as “1”) groups. GridSearch was employed to pick the proper C and gamma parameters for the SVM model. We used TCGA data for both training and testing; no separate or external validation set was used. Specifically, the numbers of training and testing samples (*n*) were 362, 246, 99, 206, 104, 87, 91, and 1335 for the LUNG, BRCA, COAD, HNSC, STAD, UCEC, LIHC, and pan-cancer cohorts, respectively (Table S4, sheet A). A total of seven features (GSVA, ssGSEA, and combined Z-score for p53tru-DR and p53tru-UR genes, respectively, and PC1 for all feature genes) were used in the SVM models. Therefore, *p* ≪ *n* for all SVM models built in this study, where *p* represents the number of features (predictor variables) and *n* represents the number of samples.

We evaluated the performance of the SVM models of individual cancer types in the following steps. Firstly, we employed the “train_test_split” function from the “sklearn.model_selection” class to partition the samples — comprising both NT and *TP53*^TM^ samples — into a training set (constituting 75% of the data) and a separate testing set (comprising the remaining 25%). Subsequently, we performed the DEA to select feature genes (p53-regulated genes) with samples from the training set. This approach ensures that the selected feature genes are based solely on the training data, providing more convincing and independent evaluation results. Finally, we built the SVM models and used the testing set to evaluate the performance. We repeated this process ten times to mitigate the potential variability in the outcomes of cross-validation. In each iteration, a confusion matrix was made, and performance measurements (*i.e.*, sensitivity/recall, precision, and accuracy) were calculated. We summarized the performance of models with the mean of the measurement scores and the receiver operating characteristic (ROC) curves. The performance measurements are defined as below:

(2) $$\begin{array}{c} \mathrm{Recall}=\frac{\mathrm{TP}}{\mathrm{TP}+\mathrm{FN}} \end{array}$$

(3) $$\begin{array}{c} \mathrm{Precision}=\frac{\mathrm{TP}}{\mathrm{TP}+\mathrm{FP}} \end{array}$$

(4) $$\begin{array}{c} \mathrm{Accuracy}=\frac{\mathrm{TP}+\mathrm{FN}}{\mathrm{TP}+\mathrm{TN}+\mathrm{FP}+\mathrm{FN}} \end{array}$$

(5) $$\begin{array}{c} F1-\mathrm{score}=\frac{2}{{\mathrm{Recall}}^{-1}+{\mathrm{Precision}}^{-1}} \end{array}$$

where FN = false negative; FP = false positive; TN = true negative; TP = true positive. We used the scikit-learn (www.scikit-learn.org) Python package for SVM modeling ten-time hold-out validation. Details of SVM models are available in Table S9. All TCGA sample barcodes for training and prediction used in this study are listed in Table S4 (sheets B–I).

### Reevaluation of *TP53* mutation statuses using RNA-seq

The genomic positions of *TP53* mutations in LUNG and BRCA samples were downloaded from the cBioPortal (https://www.cbioportal.org/). The reference and mutant allele counts were then calculated from the RNA-seq BAM file using SAMtools [77]. Samples with fewer than 10 mapped reads at a given genome site were excluded, and a cutoff of minor allele frequency = 0.1 was employed to determine the genotype. A sample was considered *TP53* mutated if it had at least five high-quality (Phred-scaled sequencing quality and mapping quality > 30) reads supporting the mutant allele. The Integrative Genomics Viewer (IGV) was used to inspect the variants manually.

### Investigation of two independent treatment-related signatures

Tumor chemotherapy sensitivity (*i.e.*, RPS) was estimated based on four genes involved in DNA repair (*RIF1*, *PARI/F2R*, *RAD51*, and Ku80/*XRCC5*) reported by Pitroda et al. [54]. Tumor RSS scores in BRCA were calculated based on a 51-gene panel reported by Speers et al. [55], and the genes were divided into “positive” and “negative” groups according to the correlations between their expression level and radiation sensitivity. The R package GSVA [34] was used to calculate the RPSs and RSS scores. Notably, according to the study of Pitroda et al. [54], the original RPS was defined as the sum of the expression levels times −1 after log_2_ transformation and robust multi-array average (RMA) normalization. In our study, RPS was represented by a negative GSVA score calculated from TPM.

### GBM-derived xenografts

GBM PDX models were generated by the Mayo Clinic Brain Tumor PDX National Resource [56]. Mice with established orthotopic tumors were randomized into groups of 5–10 mice and treated with placebo and RT. The raw sequencing data are available from the National Center for Biotechnology Information (NCBI) Sequence Read Archive (SRA: PRJNA543854 and PRJNA548556). Annotated genomic and transcriptomic data are also publicly accessible through the cBioPortal (https://www.cbioportal.org/study/summary?id=gbm_mayo_pdx_sarkaria_2019). To determine the RT responsiveness of PDXs, we calculated the ratio of median survival days between the RT group and the placebo group. A cutoff value of 1.52 was determined as the changing point on the non-parametric kernel density curve (Table S7).

## Code availability

Python source code of our p53 status prediction method is available at GitHub (https://github.com/liguowang/epage) and BioCode (https://ngdc.cncb.ac.cn/biocode/tool/BT7490).

## CRediT author statement

**Qianpeng Li:** Data curation, Formal analysis, Investigation, Methodology, Visualization, Writing – original draft, Writing – review & editing. **Yang Zhang:** Data curation, Formal analysis, Investigation, Methodology, Software, Visualization, Writing – review & editing. **Sicheng Luo:** Formal analysis. **Zhang Zhang:** Writing – review & editing. **Ann L. Oberg:** Writing – review & editing. **David E. Kozono:** Writing – review & editing. **Hua Lu:** Writing – review & editing. **Jann N. Sarkaria:** Writing – review & editing. **Lina Ma:** Conceptualization, Project administration, Writing – review & editing. **Liguo Wang:** Conceptualization, Project administration, Writing – review & editing. All authors have read and approved the final manuscript.

## Supplementary material

Supplementary material is available at *Genomics, Proteomics & Bioinformatics* online (https://doi.org/10.1093/gpbjnl/qzae064).

## Competing interests

The authors have declared no competing interests.

## Supplementary Material

qzae064_Supplementary_Data
