# Supplementary material for: Identify Non-mutational p53 Functional Deficiency in Human Cancers
Source: Genomics Proteomics Bioinformatics. 2024 Sep 26;22(5):qzae064. doi: 10.1093/gpbjnl/qzae064 (PMC11702981; doi:10.1093/gpbjnl/qzae064)
Supplement: qzae064_Supplementary_Data [file qzae064_supplementary_data.zip › SupFig5.pdf]

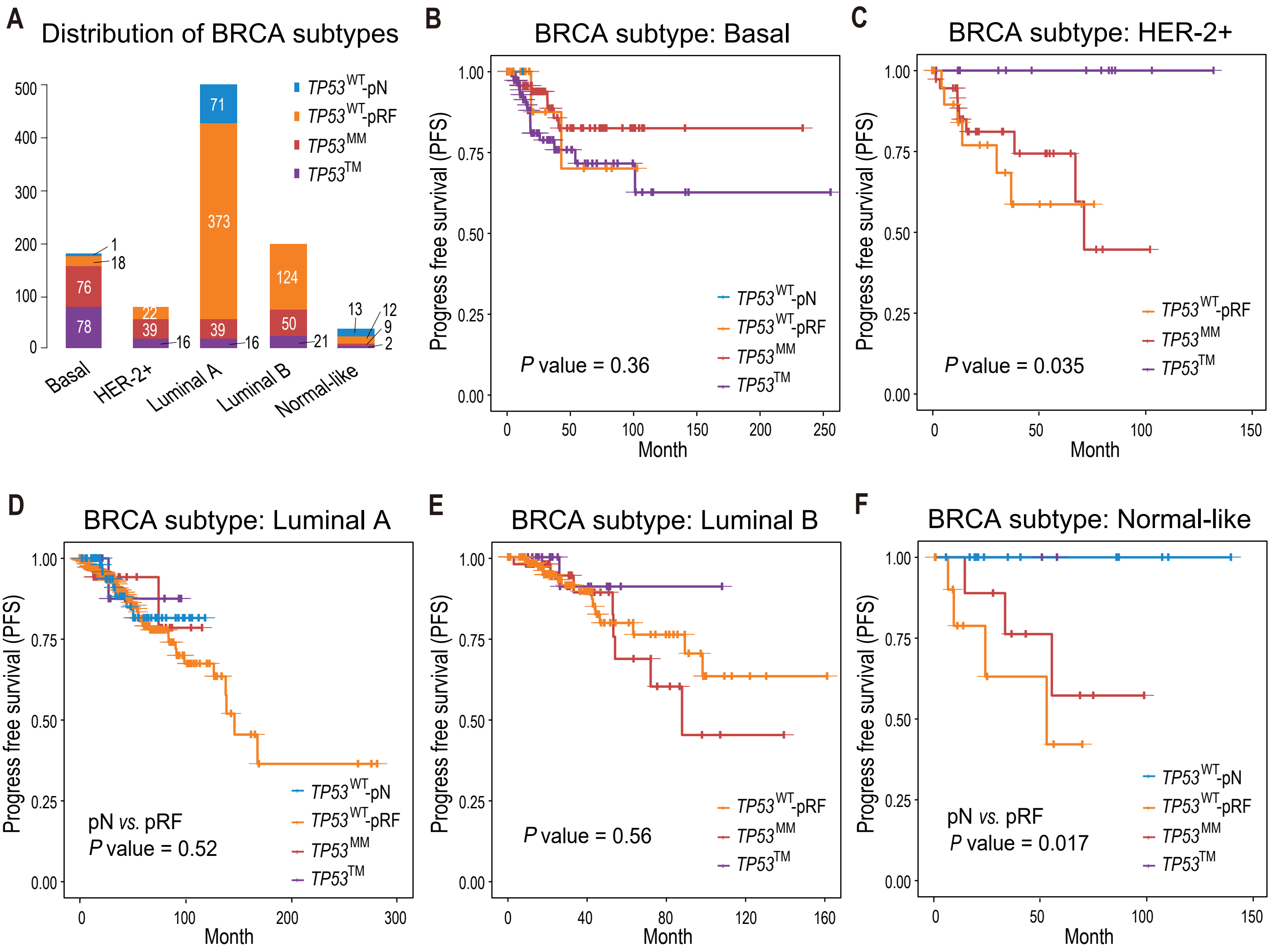
A
Distribution of BRCA subtypes
B
BRCA subtype: Basal
C
BRCA subtype: HER-2+
D
BRCA subtype: Luminal A
E
BRCA subtype: Luminal B
F
BRCA subtype: Normal-like
Basal
HER-2+
Luminal A
Luminal B
Normal-like
TP53WT-pN
TP53WT-pRF
TP53MM
TP53TM
Progress free survival (PFS)
Month
P value = 0.36
P value = 0.035
pN vs. pRF
P value = 0.52
P value = 0.56
pN vs. pRF
P value = 0.017
