# Supplementary material for: Identify Non-mutational p53 Functional Deficiency in Human Cancers
Source: Genomics Proteomics Bioinformatics. 2024 Sep 26;22(5):qzae064. doi: 10.1093/gpbjnl/qzae064 (PMC11702981; doi:10.1093/gpbjnl/qzae064)
Supplement: qzae064_Supplementary_Data [file qzae064_supplementary_data.zip › supplementary material captions-revised_09_10.docx]

Supplementary material

Figure S1 Validation of p53 target genes in γ irradiated MCF-7 cells harboring wild-type *TP53*

**A.** Changes of expression levels of p53 targets after γ irradiation, and γ irradiation with nutlin treatment. **B.** Visualization of p53 binding sites (p53 ChIP-seq signals) around the promoter regions of genes in A. hr, hours.

Figure S2 Correlations between different CESs

**A.–C.** Significant inverse correlations between CESs calculated from p53tru-DR and p53tru-UR genes for GSVA (A), ssGSEA (B), and Z-score (C). **D.–F.** Significant positive correlations between different CESs for p53tru-DR genes, GSVA *vs*. ssGSEA (D), GSVA *vs*. Z-score (E), and GSVA *vs*. PC1 (F). **G.** and **H.** Significant positive correlations between different CESs of p53tru-UR genes for GSVA *vs*. ssGSEA (G) and GSVA *vs*. Z-score (H). **I.** Significant inverse correlation between PC1 and the GSVA of p53tru-UR genes.

Figure S3 Comparison of CES distributions among different *TP53* groups in lung cancer

**A.–C**. IQR (A), variance (B), and **s**tandard deviation (C) of CESs in NT, WT, MM, and TM groups. IQR, interquartile range.

Figure S4 Pan-cancer analysis

**A.** Heatmap shows the expression profiles of p53tru-DR genes and p53tru-UR genes across 9 cancer types. **B.** ROC curve of the SVM model built from the pan-cancer cohort. **C**. CESs calculated from p53tru-DR genes and p53tru-UR genes by different algorithms in the pan-cancer cohort (similar to Figure 2). COAD, colon cancer; HNSC, head and neck cancer; STAD, stomach cancer; UCEC, endometrioid cancer; LIHC, liver cancer; BLCA, bladder urothelial carcinoma; ESCA, esophageal carcinoma;

Figure S5 PFS comparison among *TP53*^MM^, *TP53*^TM^, *TP53*^WT^-pN, and *TP53*^WT^-pRF patients of TCGA BRCA cohort

**A.** Bar plot shows the breakdown of BRCA patients into five subtypes, including “Basal/triple negative”, “HER2^+^”, “Luminal A”, “Luminal B”, and “Normal-like”. Each subtype is further divided into four subgroups based on *TP53* status. **B.–F.** Comparison of PFS for Basal (B), HER2^+^ (C), Luminal A (D), Luminal B (E), and Normal-like (F) subtype, respectively. PFS, progression-free survival.

Figure S6 Relationships between p53 status and chemotherapy and radiation therapy sensitivities after removing the overlapped genes

**A.** Venn diagram shows the overlap between p53tru-UR and p53tru-DR genes used in the LUNG and the BRCA SVM models, the chemotherapy gene signature (RPS), and the RSS. **B.** and **C.** Comparison of the RPS scores (represented by negative GSVA here, see Materials and methods) amongst NT, *TP53*^WT^-pN, *TP53*^WT^-pRF, *TP53*^MM^, and *TP53*^TM^ in the TCGA LUNG (B) and BRCA (C) cohort, respectively (similar to Figure 4A and B). **D.** and **E.** Comparison of the RSS scores of positive (D) and negative (E) genes amongst NT, *TP53*^WT^-pN, *TP53*^WT^-pRF, *TP53*^MM^-pN, *TP53*^MM^ pRF, and *TP53*^TM^ in the TCGA BRCA cohort (similar to Figure 4C and D). GSVA was used to calculate the PRS and RSS scores. Genes overlapped with p53tru-DR or p53tru-UR genes of either LUNG or BRCA were removed when calculating the RPS and RSS scores.

Figure S7 Comparison of p53 expression levels between *TP53*^WT^-pRF and *TP53*^WT^-pN samples in LUNG cohort

**A.** Comparison of the *TP53* RNA expression. **B.** Comparison of the p53 protein abundance. FPKM, fragments per kilobase of exon per million mapped fragments; RBN, replicates-based normalization; RPPA, reverse phase protein array.

Figure S8 Examples of *TP53* somatic mutations identified from RNA-seq data but missed by TCGA WES calls

MAF is measured by the ratio between the “number of reads supporting the mutant allele” and the “total number of reads” covering the mutation site. MAF, mutant allele fraction; WES, whole-exome sequencing.

Figure S9 p53 mutants identified from RNA-seq data exhibit similar genomic characteristics as the *TP53*^MM^ and *TP53*^TM^ groups

**A.–C.** Compared to the *TP53*^WT^-pN tumors, tumors of the RM group show increased tumor mutation burden (A), copy number variation burden (B), aneuploidy score (C), and Buffa hypoxia score (D). RM, p53 mutants identified from RNA-seq data.

Table S1 List of p53 target genes used in this study

Table S2 CESs of p53tru-DR and p53tru-UR genes in LUNG, BRCA, COAD, HNSC, STAD, UCEC, LIHC, and pan-cancer (including 9 types or subtypes) cohorts

Table S3 The performance metrics of the SVM model in TCGA LUNG, BRCA, COAD, HNSC, STAD, UCEC, LIHC, and pan-cancer cohorts

Table S4 List of SVM training samples, prediction samples, *TP53* original genetic states, SVM-predicted states, and probabilities of TCGA cancers analyzed in our study

Table S5 Genomic and clinical characteristics of TCGA LUNG, BRCA, COAD, HNSC, LIHC, STAD, UCEC, and pan-cancer cohorts

Table S6 Gene signatures reflecting chemosensitivity or radiosensitivity

Table S7 Median survival days and CES values of PDX models treated with placebo and radiation therapy

Table S8 List of lung and breast cancer samples that have *TP53* missense mutations detected from RNA-seq

Table S9 Summary of SVM models according to the DOME recommendations
