# Supplementary figures and images for: Identify Non-mutational p53 Functional Deficiency in Human Cancers
Source: Genomics Proteomics Bioinformatics. 2024 Sep 26;22(5):qzae064. doi: 10.1093/gpbjnl/qzae064 (PMC11702981; doi:10.1093/gpbjnl/qzae064)

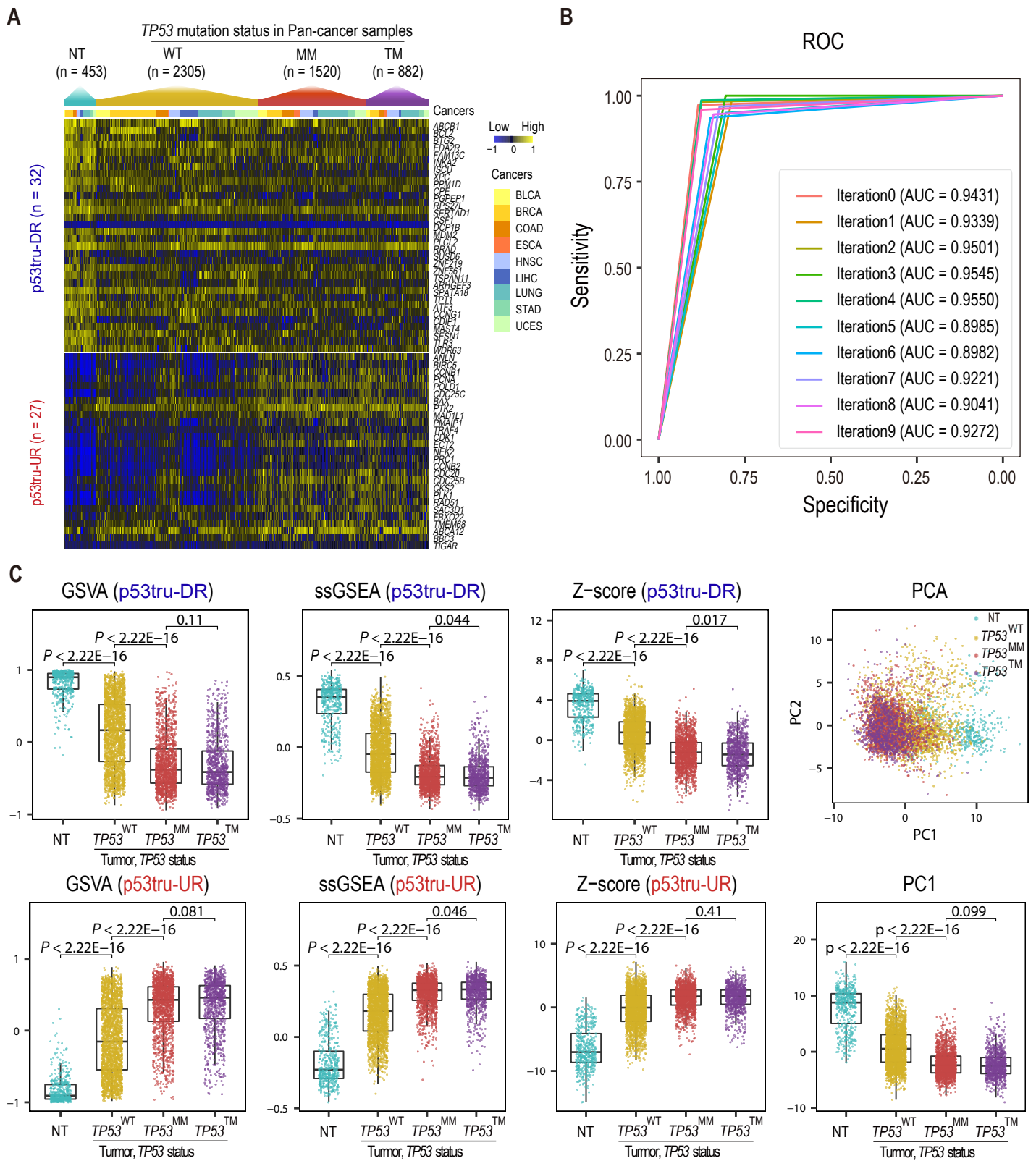

Supplement: qzae064_Supplementary_Data [file qzae064_supplementary_data.zip › SupFig4.pdf]

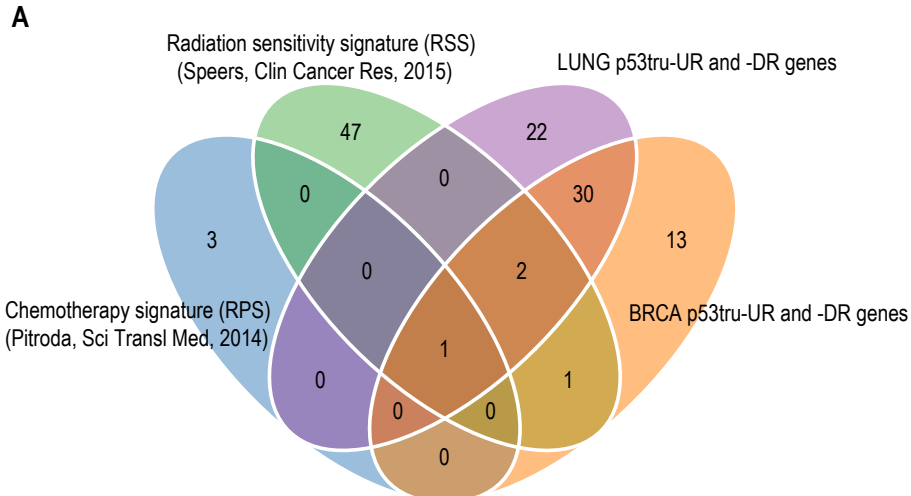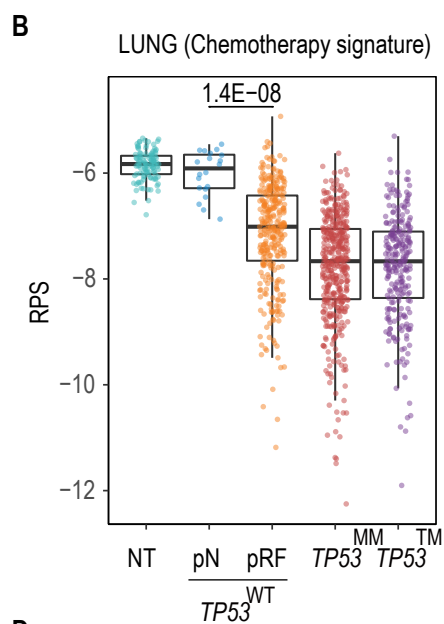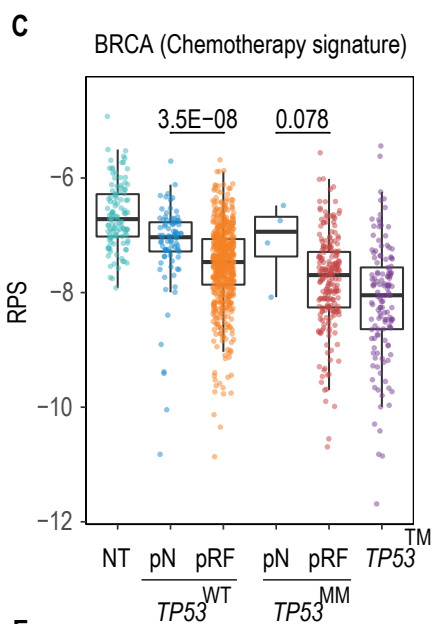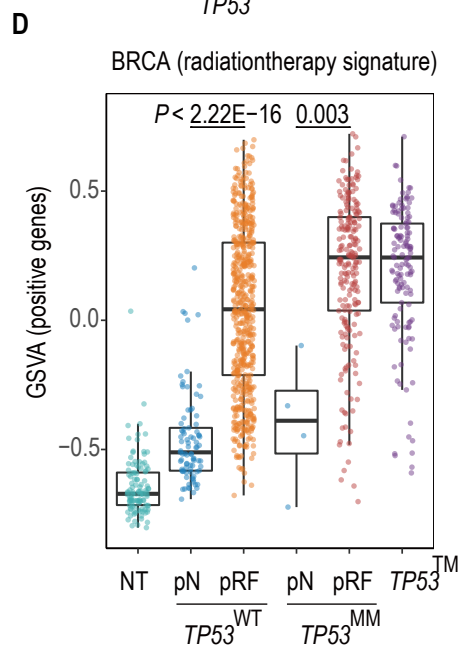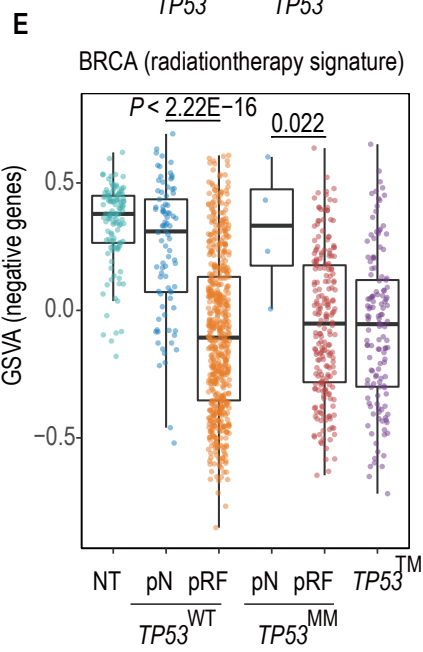

Supplement: qzae064_Supplementary_Data [file qzae064_supplementary_data.zip › SupFig6.pdf]

**A***TP53* RNA expression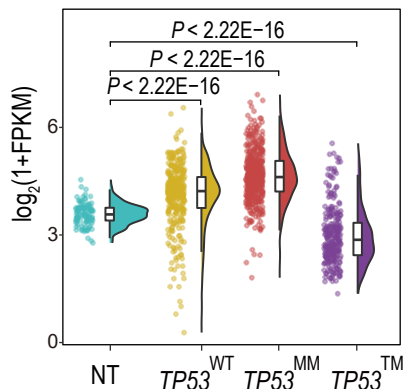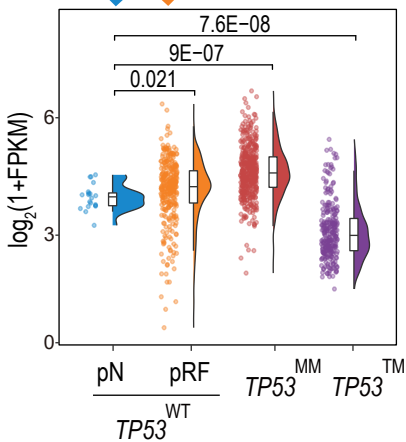**B**

p53 protein expression

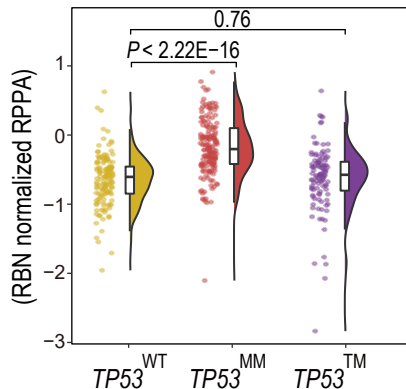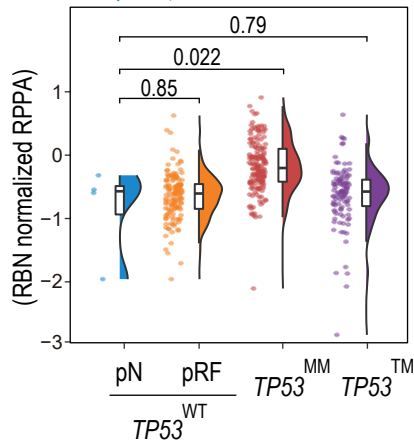

Supplement: qzae064_Supplementary_Data [file qzae064_supplementary_data.zip › SupFig7.pdf]

A

TCGA-55-6987

RNA-seq (hg19)

WES (hg38)

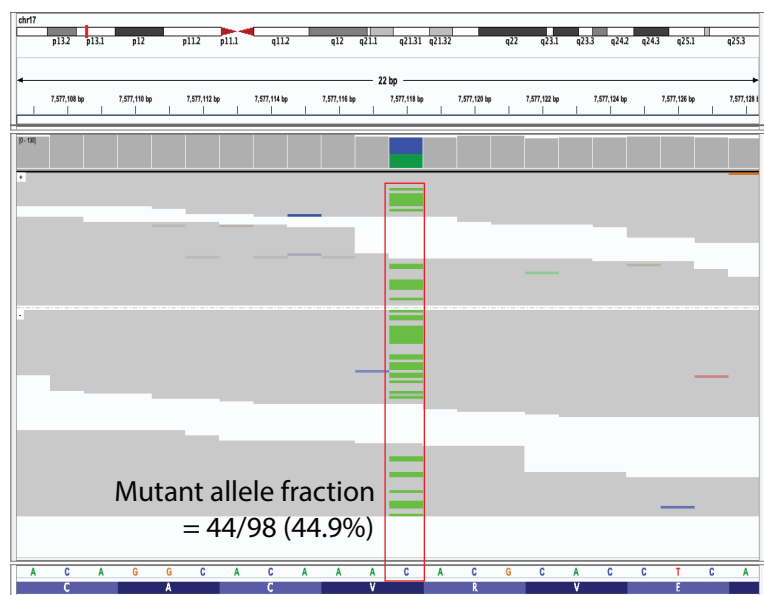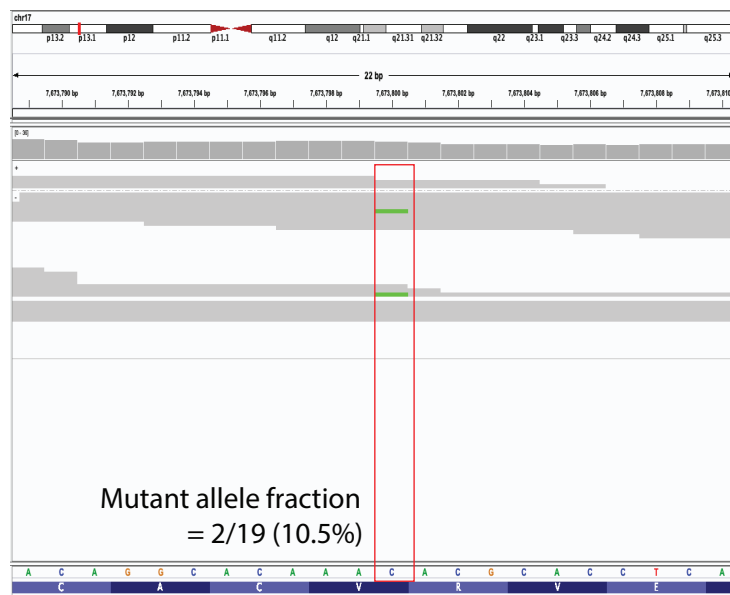

B

TCGA-55-8621

RNA-seq (hg19)

WES (hg38)

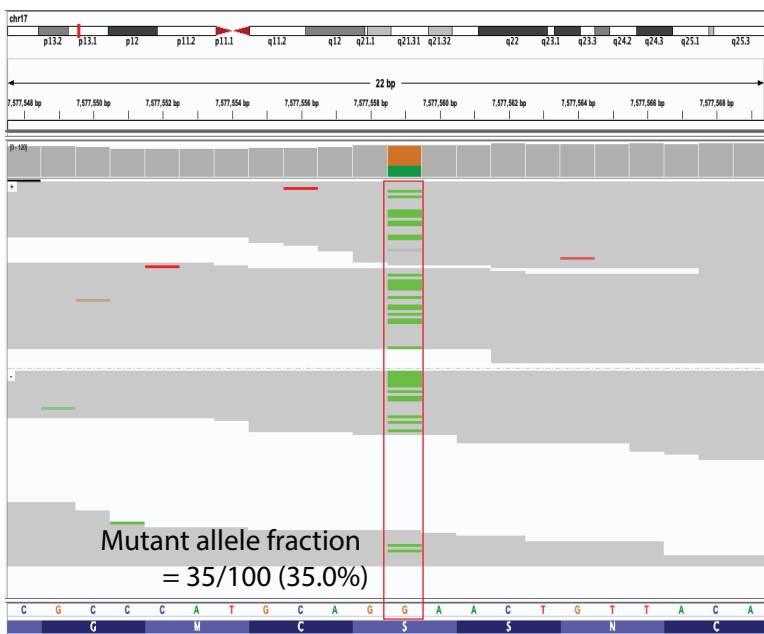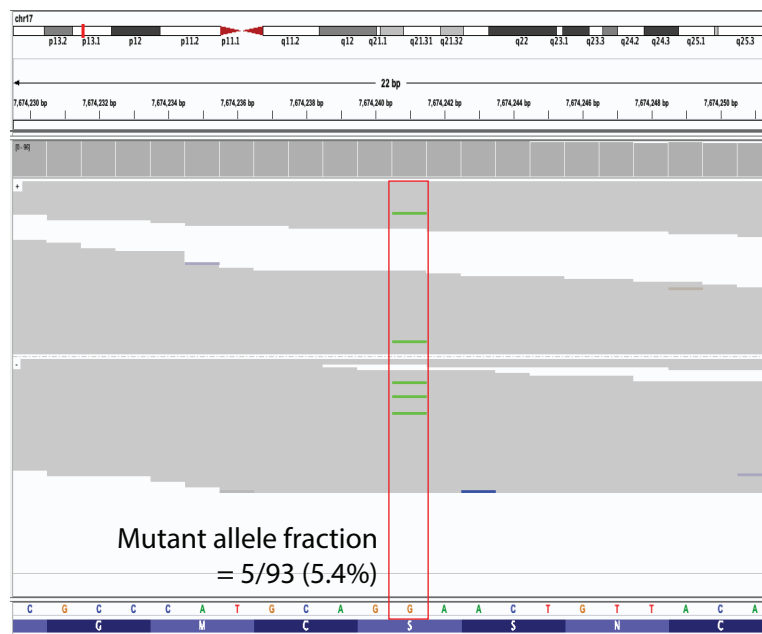

Supplement: qzae064_Supplementary_Data [file qzae064_supplementary_data.zip › SupFig8.pdf]

**A**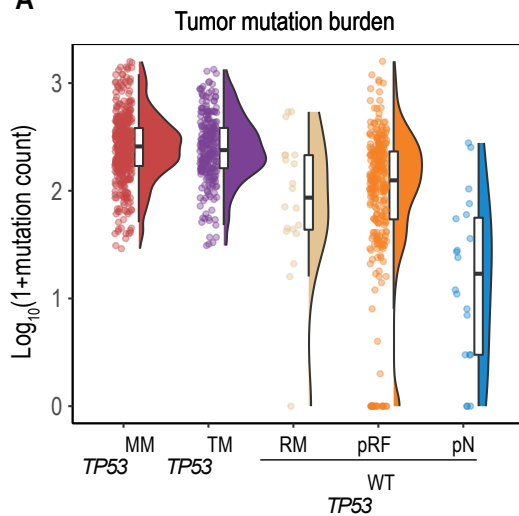**B**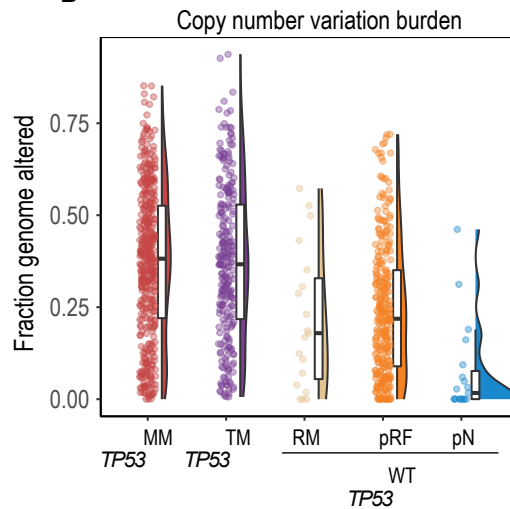**C**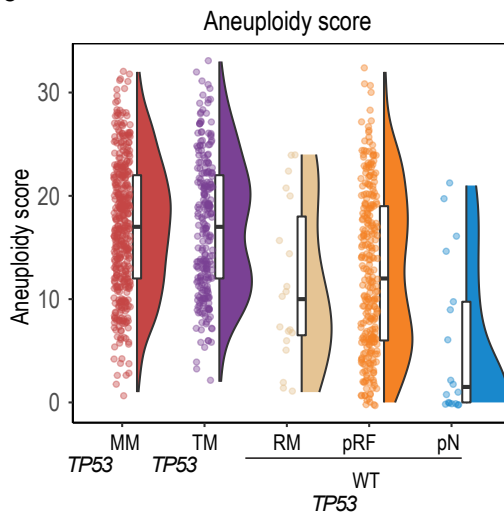**D**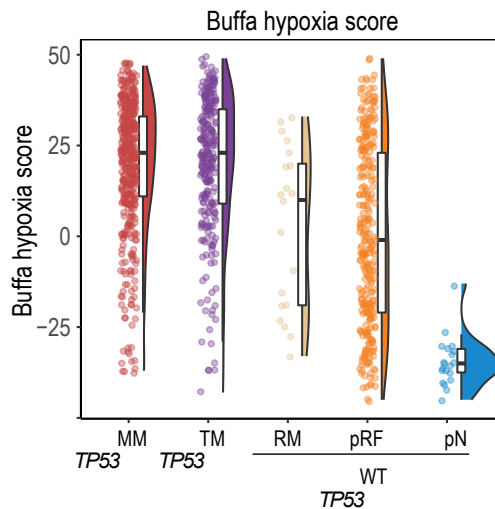

Supplement: qzae064_Supplementary_Data [file qzae064_supplementary_data.zip › SupFig9.pdf]

A

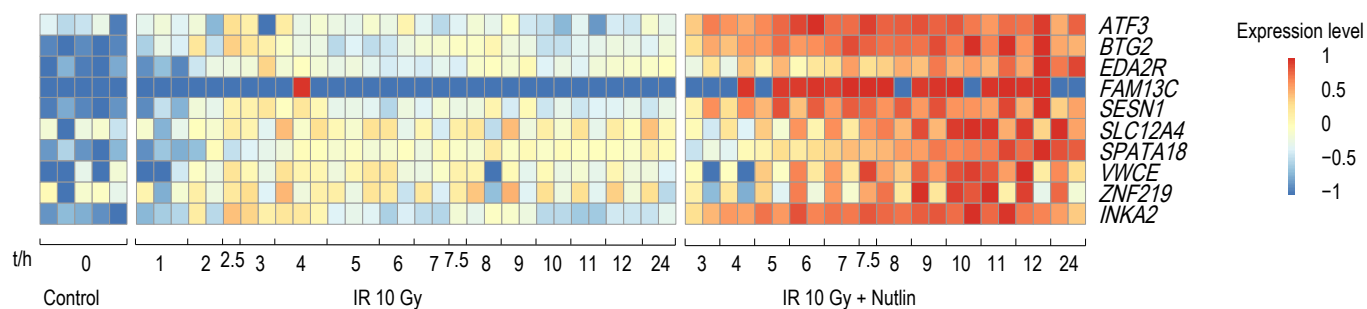

B

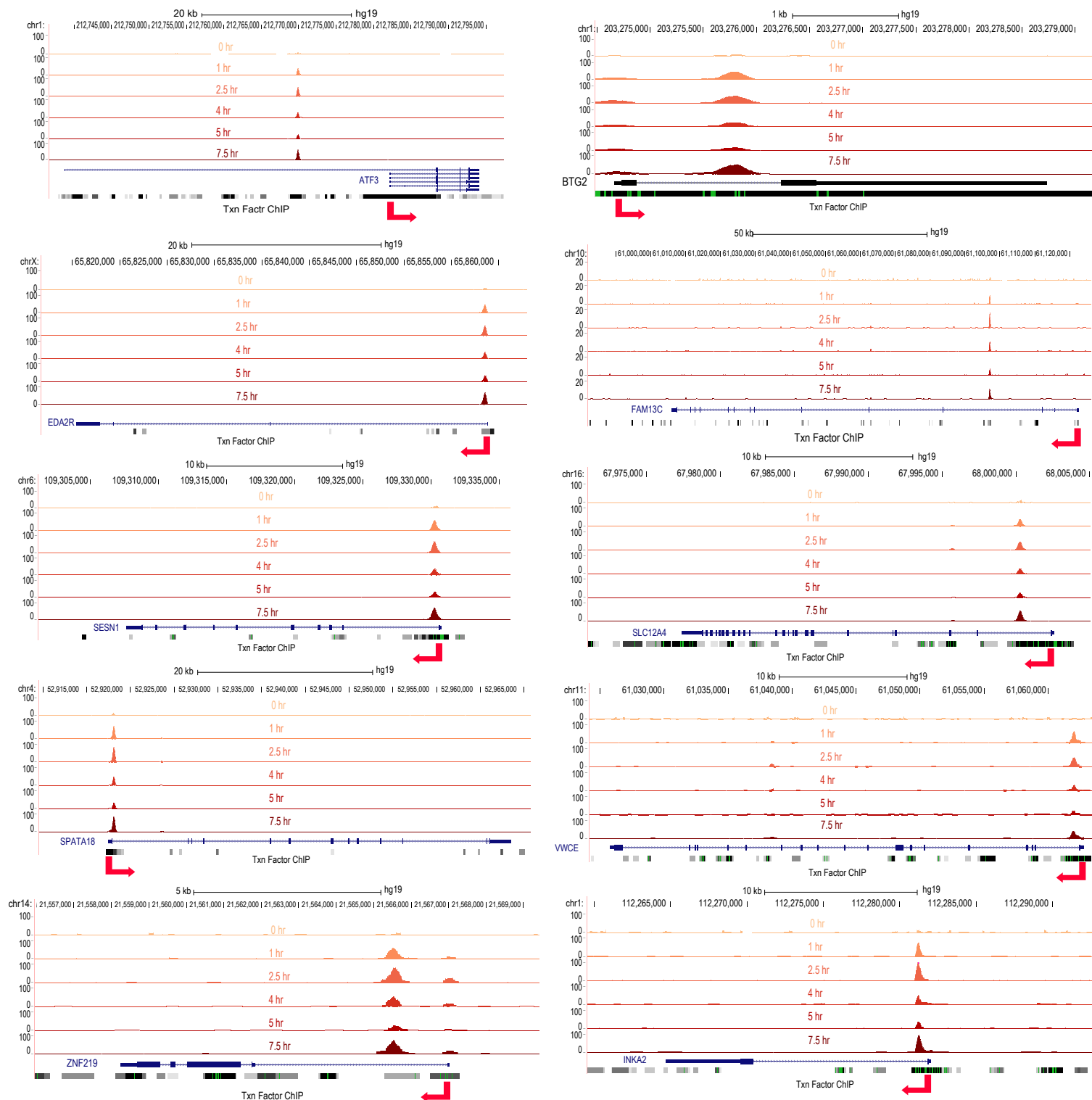

Supplement: qzae064_Supplementary_Data [file qzae064_supplementary_data.zip › SupFig1.pdf]

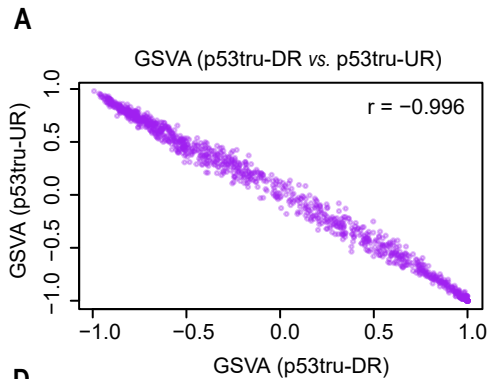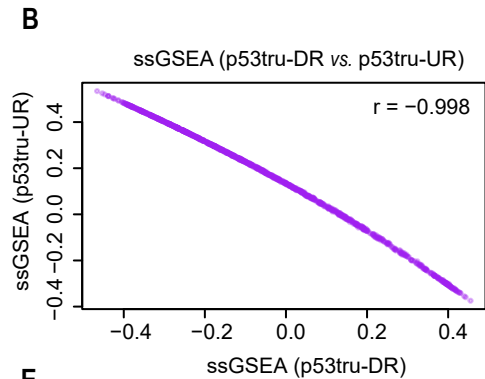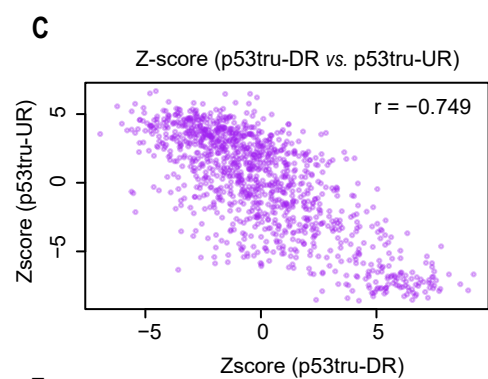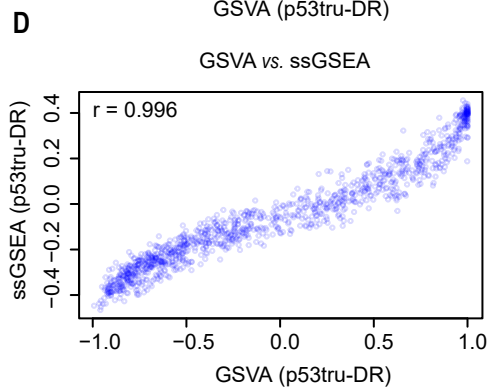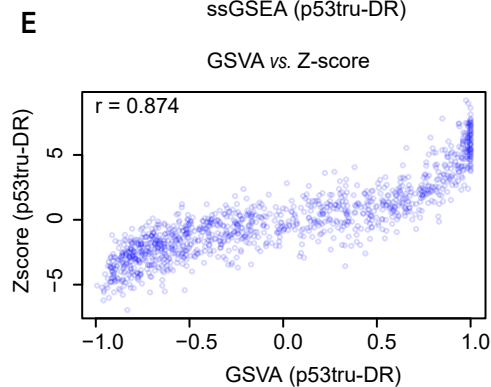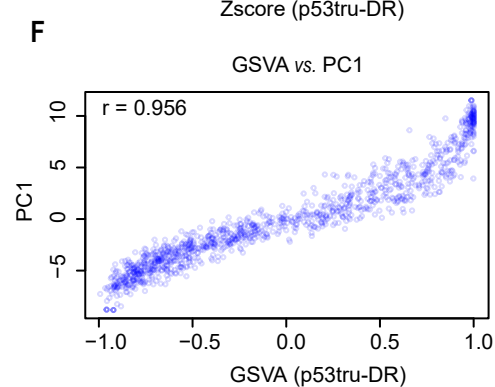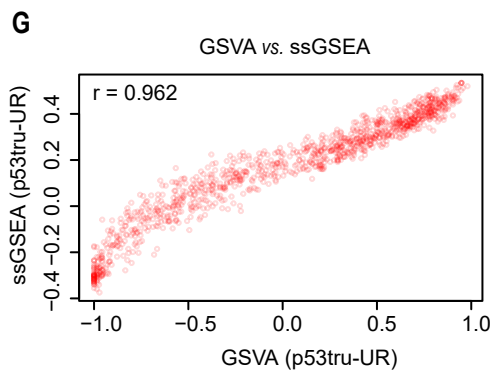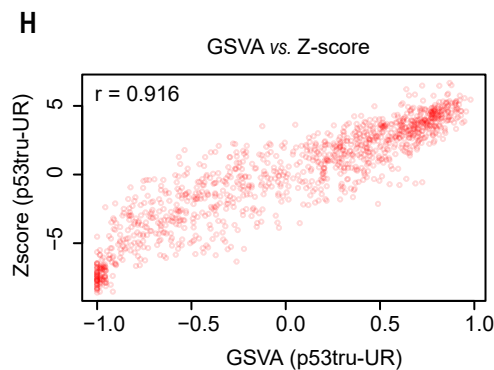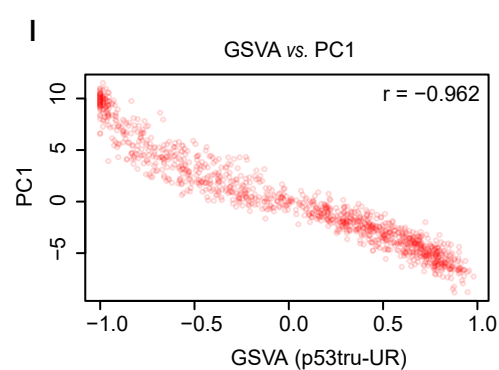

Supplement: qzae064_Supplementary_Data [file qzae064_supplementary_data.zip › SupFig2.pdf]

**A**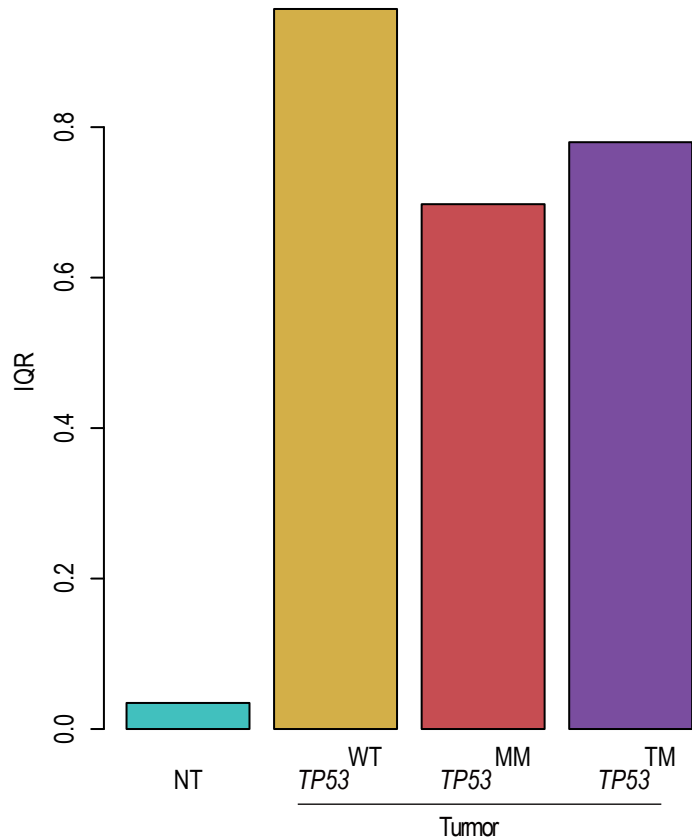**B**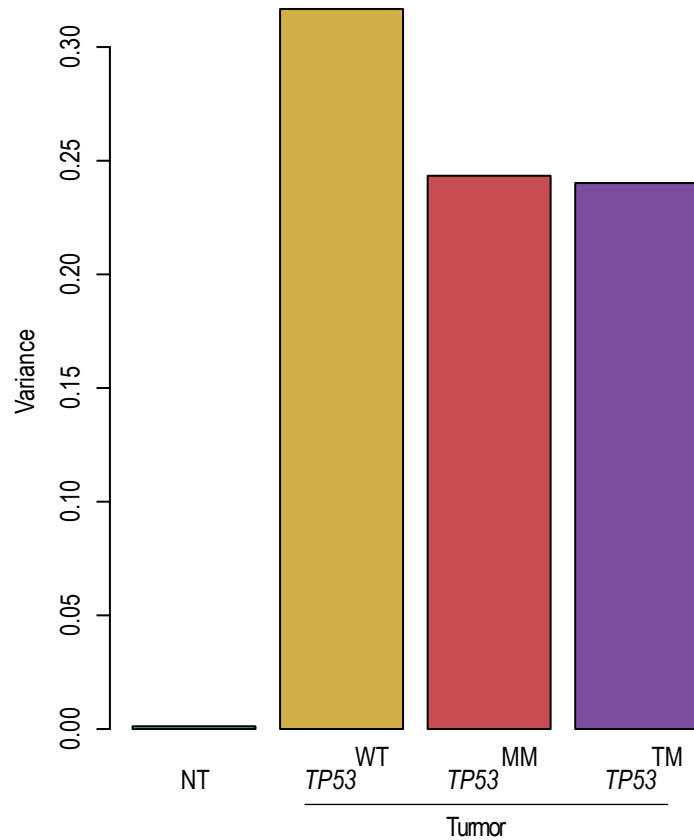**C**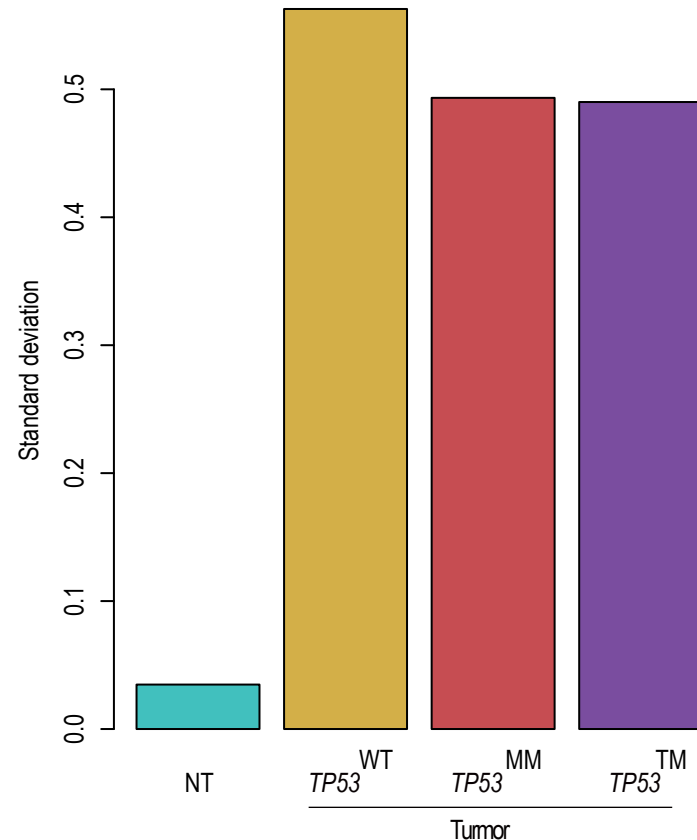

Supplement: qzae064_Supplementary_Data [file qzae064_supplementary_data.zip › SupFig3.pdf]
